# Machine learning and Mendelian randomization identify key lifestyle factors in coronary heart disease: A NHANES-Based study

**DOI:** 10.1016/j.ijcrp.2025.200536

**Published:** 2025-10-26

**Authors:** Yang yang Cui, Yonghong Zhang, Lang Zeng, Shikang Li, Xue Mei, Xiangmei Yang, Peng Zhou, Lijuan Xiong, Yijuan Huang, Jing Luo, Fenglin Wu, Rongchuan Yue

**Affiliations:** a Department of Cardiology, Affiliated Hospital of North Sichuan Medical College, Nanchong, 637000, China; b Department of Cardiology, People's Hospital of Guang 'an District, Guang 'an, 638550, China; c School of Pharmacy, Institute of Material Medica, North Sichuan Medical College, Sichuan, Nanchong, 637000, China; d Department of Emergency, Affiliated Hospital of North Sichuan Medical College, Nanchong, 637000, China

**Keywords:** NHANES, Machine learning, Mendelian randomization, Coronary heart disease, Causal inference

## Abstract

**Objective:**

This study aims to bridge the gap between predictive modeling and causal inference by utilizing lifestyle data from the National Health and Nutrition Examination Survey (NHANES) database to compare the predictive performance of multiple machine learning models for coronary heart disease (CHD). By incorporating Mendelian randomization, the study seeks to validate and identify the lifestyle variables with both predictive power and causal impact on CHD.

**Methods:**

We extracted variables related to demographic characteristics and lifestyle from the NHANES database (2013–2018; n = 29,400). Recursive feature elimination (RFE) was employed to rank variable importance and determine the optimal feature subset. Subsequently, eight machine learning models-including Support Vector Machine (SVM), Neural Network (NN), Naive Bayes (NB), Extreme Gradient Boosting (XGBoost), Multilayer Perceptron (MLP), Generalized Linear Model (GLM), Adaptive Boosting (AdaBoost), and Decision Tree (DT)-were developed for CHD prediction. Model performance was evaluated using metrics such as accuracy, precision, sensitivity, specificity, recall, F1-score, and the Receiver Operating Characteristic (ROC) curve, with variable contributions visualized using Shapley Additive Explanations (SHAP). Additionally, Mendelian randomization (MR) was applied to distinguish associative from causal relationships, validating top predictors via Genome-Wide Association Study (GWAS)-derived genetic instruments.

**Results:**

RFE identified age, sex, fasting blood glucose, body mass index (BMI), total cholesterol (TC) intake, sleep duration, diastolic blood pressure, and smoking as the most significant predictors of CHD. Among the models, SVM outperformed DT, AdaBoost, XGBoost, NN, MLP, NB, and GLM. The SVM model achieved the highest performance with an accuracy of 83.4 % and an AUC value of 0.909, demonstrating clinically actionable predictive power. MR confirmed causal associations for five variables: BMI (OR: 1.01, *P* < 0.001), TC (OR: 1.01, *P* < 0.001), insomnia (OR: 1.03, *P* < 0.001), diastolic blood pressure (OR: 1.20, *P* < 0.001), and smoking (OR: 1.03, *P* < 0.001), while fasting glucose showed null causality (*P* > 0.05).

**Conclusion:**

The SVM machine learning model, based on NHANES data, enables faster and more efficient prediction of CHD. The study identified age, sex, BMI, TC intake, sleep duration, diastolic blood pressure, and smoking as the lifestyle variables with the greatest impact on CHD. This dual approach advances precision prevention by combining predictive accuracy with genetic evidence.

## Highlights

• This study integrates machine learning and Mendelian randomization to improve coronary heart disease (CHD) risk prediction and causal inference using NHANES lifestyle data.
• Mendelian randomization confirmed causal effects for five lifestyle factors, strengthening the evidence beyond association for BMI, cholesterol intake, sleep duration, diastolic blood pressure, and smoking.
• This integrated approach enhances precision prevention of CHD by combining robust predictive modeling with genetic validation of causal risk factors.

## 1 Introduction

Coronary heart disease (CHD), primarily driven by coronary atherosclerosis, remains a leading global cause of mortality despite advances in diagnosis and treatment [1]. Its rising prevalence is closely linked to population aging and unfavorable shifts in lifestyle and dietary patterns [2]. There is an urgent need for innovative strategies to effectively mitigate its prevalence, particularly through enhanced public health campaigns and the promotion of healthier lifestyles. A decade-long cohort study utilizing the UK Biobank database revealed a significant association between frequent night shift work and an increased risk of CHD, with a hazard ratio (HR) of 1.22 (95 % confidence interval: 1.11–1.35) [3]. Sedentary behavior, obesity, and smoking have also been shown to significantly increase CHD incidence, while higher red meat consumption has been positively correlated with disease risk [4,5]. Although these findings underscore the multifaceted role of lifestyle—encompassing diet, physical activity, sleep, and substance use—in CHD development, observational evidence is inherently limited by confounding and reverse causation. Establishing causal relationships between specific lifestyle factors and CHD risk therefore requires more robust analytical approaches.

Machine learning is increasingly used in medicine for high-dimensional prediction and feature selection [6,7]. In cardiovascular research, gradient-boosted trees and multi-layer perceptrons reached 95 % accuracy for CHD prediction, echoing the 95 % performance recently reported for deep-learning systems that use high-risk treadmill ECG features to detect obstructive CAD and for networks that forecast short-term mortality in acute pulmonary embolism [[8], [9], [10]]. These studies show that machine learning can both quantify CHD risk and flag its principal drivers. However, population-based models that predict CHD from lifestyle data rarely include sleep metrics and remain genetically untested; moreover, their associations are confounded and causally unverified [11,12]. Integrating prediction with Mendelian randomization addresses these gaps.

**Fig. 1:**
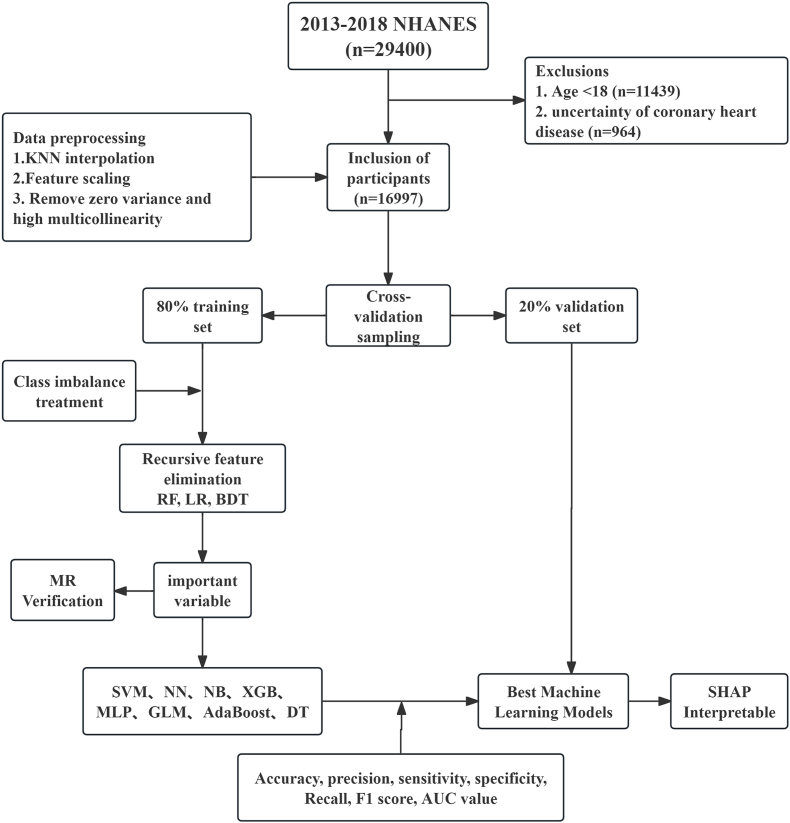
**Study design flow** The flow shows the process of screening and machine learning the NHANES database data, including the validation of Mendelian randomization.

We integrate machine learning with two-sample MR to unify prediction and causal testing. In 20,000+ NHANES adults we train and tune eight lifestyle-only models for incident CHD, select the best on nested cross-validation, then verify the top predictors by MR with publicly available GWAS summary statistics (Fig. 1). This yields an accurate, biomarker-free screening tool and a genetically supported shortlist of modifiable targets for precision prevention.

## 2 Study population and methods

### 2.1 Machine learning

#### 2.1.1 Data source and participants

We used the 2013–2018 NHANES cycles (n = 29,400), a cross-sectional, nationally representative survey conducted by the CDC. NHANES records self-reported physician diagnoses of CHD, lifestyle, diet, and laboratory data; all participants gave informed consent and the NCHS Research Ethics Review Board approved the protocol [13].

#### 2.1.2 Data preprocessing

The inclusion criteria were as follows: (1) participants aged ≥18 years; (2) participants who completed the questionnaire regarding whether a doctor had informed them of having coronary heart disease (CHD), with complete questionnaire content and data. The exclusion criteria were: (1) participants aged <18 years; (2) participants with a CHD status of 9 (indicating an uncertain CHD status) based on the NHANES questionnaire [14]. Ultimately, 16,997 participants were included in the analysis. We incorporated the latest cardiovascular health metrics updated by the American Heart Association—Life's Essential 8 (LE8) [15]—and included 21 variables from four domains: (1) Demographic data (age, gender, education level, race, marital status); (2) Questionnaire data (physical activity, sleep status, smoking, and alcohol consumption); (3) Examination data (body mass index [BMI], poverty index, fasting blood glucose, systolic and diastolic blood pressure); and (4) Dietary data (energy, protein, carbohydrates, total sugar, dietary fiber, total cholesterol [TC], and total fat). The diagnosis of CHD was based on participants' self-reported questionnaire data indicating that a doctor had informed them of having CHD. All analyses were performed without sample weights because the aim was to examine biological associations rather than produce nationally representative estimates.

We first conducted a statistical analysis of missing values and chose the K-Nearest Neighbors (KNN) imputation method (with K = 5) to complete the dataset. This method is more accurate than simple imputation techniques (e.g., mean or median imputation), especially for small to medium-sized datasets [16]. To meet the data format requirements of machine learning algorithms, we normalized the data to a range of (0, 1) using feature scaling [17]. We removed variables with zero variance and those exhibiting high multicollinearity to enhance model performance, interpretability, and reduce the risk of overfitting [18]. The dataset was divided using K-fold cross-validation with K = 5, resulting in 4 training sets and 1 test set. The final training set contained 13,598 samples, and the validation set contained 3399 samples [19]. To address the significant disparity between positive (CHD) and negative (non-CHD) outcomes, we employed the Random Over-Sampling Examples (ROSE) method to balance the outcome variable, thereby reducing the numerical gap [[20], [21], [22]]. We utilized three common recursive feature elimination (RFE) algorithms-Random Forest (RF), Logistic Regression (LR), and Bagging Decision Trees (BDT)-to rank the importance of variables. These algorithms evaluate feature importance by iteratively building models and eliminating less important features, ultimately identifying the optimal feature subset [23].

#### 2.1.3 Machine learning framework

The dataset was divided into 80 % for training (n = 13,598) and 20 % for testing (n = 3399). We employed eight different machine learning models to identify CHD caused by lifestyle exposures: Support Vector Machine (SVM), Neural Network(NN), Naive Bayes (NB), XGBoost, Multilayer Perceptron (MLP), Generalized Linear Model (GLM), Adaptive Boosting (AdaBoost), and Decision Tree (DT). Each machine learning algorithm has its unique characteristics, as described below: SVM is a maximum-margin classifier whose computational complexity grows polynomially with both the number of features and samples [24]. NN is a multilayered, non-linear model that captures high-dimensional interactions by mimicking neuronal signal transmission [25]. NB is a Bayes-theorem-based probabilistic classifier that assumes conditional independence of features; it is computationally light and scales well to high-dimensional data [26]. XGBoost is a gradient boosting algorithm that can handle imbalanced data by adjusting weights. It is widely used for classification problems [27]. MLP is a feedforward artificial neural network that effectively captures nonlinear relationships, making it an ideal choice for classifying complex and multifactorial diseases [28]. GLM is a flexible extension of linear regression that models non-normal dependent variables by linking predictors to outcomes. It can represent binary and count outcomes through different conditional distributions and functional relationships [29]. AdaBoost is a boosting method that typically achieves high training accuracy. However, it may reduce classification accuracy for imbalanced data and increase computational complexity [30]. 10.13039/100001588DT is a A nonparametric supervised learning method that is easy to understand and interpret, supports visual analysis, but is prone to overfitting [31].

#### 2.1.4 Model evaluation

To evaluate and compare the predictive performance of the machine learning models, we employed various evaluation metrics, including: Accuracy and 95 % Confidence Interval (95 % CI), Precision, Sensitivity, Specificity, Recall, F1 Score, ROC Curve and AUC [32]. In addition to the aforementioned evaluation metrics, we introduced a confusion matrix to provide a more intuitive visualization of the predictions made by the machine learning models.

#### 2.1.5 Model optimization

The best-performing model was re-trained with an exhaustive grid-search of candidate hyper-parameters; each configuration was evaluated by 10-fold cross-validation to stabilise performance estimates. Calibration plots (predicted vs. observed probability) and decision-curve analysis were then generated to confirm that the final model supplies both well-calibrated probabilities and net clinical benefit across the range of threshold probabilities encountered in practice.

#### 2.1.6 Statistical methods

In this study, data selection, the establishment of machine learning models, model optimization, and the creation of key visualizations were conducted using R version 4.4.2. A seed number of 1234 was set for all analyses to ensure reproducibility.

### 2.2 Mendelian randomization

#### 2.2.1 Concept introduction

Large-scale genome-wide association studies conducted over the past decade have identified numerous genetic variations associated with cardiac metabolic traits and risk factors. These findings have enabled the application of Mendelian Randomization (MR), a method that uses genetic variations as instrumental variables for causal inference between exposure and outcome, thereby determining whether the presumed risk factors might have a causal impact on disease [33]. Based on the selection of variables that significantly influence CHD from the optimal machine learning models, this study attempts to utilize Mendelian Randomization to validate the causal relationships, thereby enhancing its persuasive power and clinical applicability.

#### 2.2.2 GWAS data collection and processing

Samples were obtained by accessing the IEU OpenGWAS project (mrcieu.ac.uk), collecting Genome-Wide Association Study (GWAS) data for the important variables identified through machine learning. The data for the exposure variables were preprocessed first, applying a threshold of *P* < 10^−8^ to filter single nucleotide polymorphisms (SNPs) significantly associated with the exposure variables. To ensure independence among the SNPs and to avoid the confounding effects of linkage disequilibrium, a linkage disequilibrium coefficient (r^2^ = 0.001) was established, limiting the correlation among adjacent SNPs. Additionally, to restrict the analysis scope, a region width of 10,000 kb was set, including only those SNPs within a certain distance from the target SNP, thereby excluding the influence of pleiotropy on the results to obtain instrumental variables related to the exposure variables [34]. After merging the exposure and outcome factors, a threshold of *P* < 10^−8^ was again applied to exclude SNPs that were directly associated with the outcome variable, CHD. The final dataset thus formed constitutes the requisite instrumental variables for further analysis and research in this study [35](Supplementary Table 1).

#### 2.2.3 Mendelian randomization analysis

This study employed five regression models for Mendelian Randomization: MR-Egger regression, weighted median estimation (WME), inverse variance weighting (IVW), a simple model, and a weighted model. SNPs were used as instrumental variables to validate the causal relationship between the exposure variables and the outcomes. MR-Egger regression is a causal inference method that estimates the causal effect based on instrumental variables while accounting for potential instrument bias. The WME is another non-parametric approach that can reduce potential biases by excluding data based on the median. IVW estimates the overall effect by averaging the weights based on the variance of each study. The simple model is a linear regression method that establishes relationships by either weighting or not weighting the data, aiming to estimate the genetic correlation effect. The weighted model provides more accurate estimates by considering the sample size and error variance of each study.

#### 2.2.4 Sensitivity analysis

Heterogeneity across SNPs was quantified with Cochran's Q (P < 0.05 indicates significant heterogeneity); when present, random-effects IVW was prioritised [36]. Directional horizontal pleiotropy was examined with the MR-Egger intercept [37]. Robustness was further assessed by leave-one-out analysis: exclusion of any single SNP should not materially alter the causal estimate [38].

#### 2.2.5 Statistical methods

In this study, data import, instrumental variable selection, Mendelian Randomization analysis, and sensitivity analysis were all conducted using the TwoSampleMR package within R version 4.4.2. Key parameters and assumptions were adjusted to explore variations in the results. Additionally, a significance level of *α* = 0.05 was adopted throughout the analysis.

## 3 Results

### 3.1 Machine learning component

#### 3.1.1 Baseline characteristics of NHANES participants

As is shown in Table 1, the characteristics of study participants with and without CHD from the NHANES conducted between 2013 and 2018 were summarized. A total of 16,997 participants were included in the analysis, of which 48.1 % were male, with an average age of 50.0 years. Among the participants, 741 individuals were diagnosed with CHD (4.5 %). Notably, CHD patients were predominantly male, older, and primarily non-Hispanic white compared to controls (all *P* < 0.05).

**Table 1:** Baseline table of participants.

	[ALL]	0	1	p.overall
	*N=16,997*	*N=16,256*	*N=741*	
Age	50.0 (17.7)	49.1 (17.5)	68.7 (10.7)	<0.001
Gender:				<0.001
Male	8169 (48.1 %)	7678 (47.2 %)	491 (66.3 %)	
Female	8828 (51.9 %)	8578 (52.8 %)	250 (33.7 %)	
Edu:				0.003
<High school	1612 (9.50 %)	1519 (9.36 %)	93 (12.6 %)	
Completed high school	2095 (12.3 %)	1991 (12.3 %)	104 (14.1 %)	
>High school	13,267 (78.2 %)	12,724 (78.4 %)	543 (73.4 %)	
Race:				<0.001
Mexican American	2486 (14.6 %)	2419 (14.9 %)	67 (9.04 %)	
Other Hispanic	1789 (10.5 %)	1726 (10.6 %)	63 (8.50 %)	
Non-Hispanic White	6242 (36.7 %)	5819 (35.8 %)	423 (57.1 %)	
Non-Hispanic Black	3664 (21.6 %)	3552 (21.9 %)	112 (15.1 %)	
Other Race	2816 (16.6 %)	2740 (16.9 %)	76 (10.3 %)	
Marital:				0.078
Married/Living with partner	10,053 (59.2 %)	9638 (59.3 %)	415 (56.0 %)	
Widowed/Divorced/Separated/Never married	6933 (40.8 %)	6607 (40.7 %)	326 (44.0 %)	
PIR	2.49 (1.62)	2.50 (1.63)	2.33 (1.49)	0.003
BMI	29.5 (7.21)	29.5 (7.24)	30.2 (6.66)	0.008
Smoke:				<0.001
No	9775 (57.5 %)	9497 (58.4 %)	278 (37.5 %)	
Yes	7222 (42.5 %)	6759 (41.6 %)	463 (62.5 %)	
Alcohol:				0.930
No	12,957 (90.6 %)	12,385 (90.6 %)	572 (90.8 %)	
Yes	1342 (9.39 %)	1284 (9.39 %)	58 (9.21 %)	
Sleep	7.43 (2.35)	7.42 (2.26)	7.69 (3.81)	0.062
Physical_Activity:				0.011
Inactive	9961 (58.6 %)	9504 (58.5 %)	457 (61.7 %)	
Moderate	3514 (20.7 %)	3349 (20.6 %)	165 (22.3 %)	
Vigorous	728 (4.28 %)	708 (4.36 %)	20 (2.70 %)	
Both moderate and vigorous	2794 (16.4 %)	2695 (16.6 %)	99 (13.4 %)	
Systolic	125 (18.9)	125 (18.6)	133 (22.3)	<0.001
Diastolic	70.6 (13.1)	70.8 (12.9)	65.7 (14.8)	<0.001
FBG	6.20 (2.11)	6.15 (2.04)	7.20 (3.03)	<0.001
Energy	2102 (1007)	2110 (1012)	1922 (857)	<0.001
Protein	81.0 (44.0)	81.3 (44.2)	72.8 (37.0)	<0.001
Carbohydrate	249 (126)	250 (127)	229 (112)	<0.001
Sugars	107 (76.5)	107 (76.6)	101 (72.8)	0.026
Dietary_fiber	17.0 (10.9)	17.0 (10.9)	16.1 (10.6)	0.040
Fat	82.5 (48.3)	82.7 (48.5)	77.4 (44.5)	0.004
Cholesterol	305 (250)	306 (252)	278 (210)	0.001

† “Cholesterol” in NHANES 2013–2018 denotes dietary cholesterol estimated from 24-h dietary recalls; it does not represent serum total cholesterol.

#### 3.1.2 Preliminary selection of important variables

**Supplementary Fig. 1:**
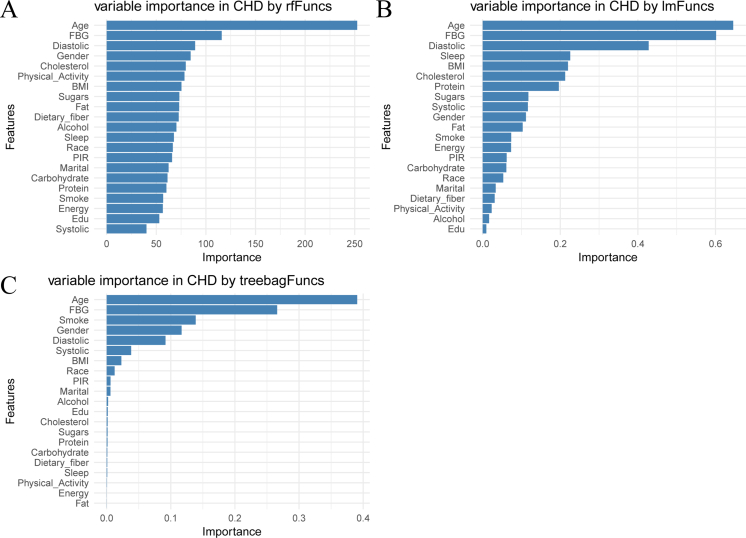
**Feature recursion analysis** The analysis shows feature selection using different algorithms: (A) RF algorithm, (B) LM algorithm, and (C) BDT algorithm.

The results of the recursive feature elimination algorithm for the ranking of important variables are presented in Supplementary Fig. 1. After synthesizing the top five important variables from three different algorithms, eight significant variables were identified for further analysis in the machine learning models. These variables include age, sex, fasting blood glucose, BMI, TC, sleep duration, diastolic blood pressure, and smoking status.

#### 3.1.3 Establishment and validation of machine learning models

**Fig. 2:**
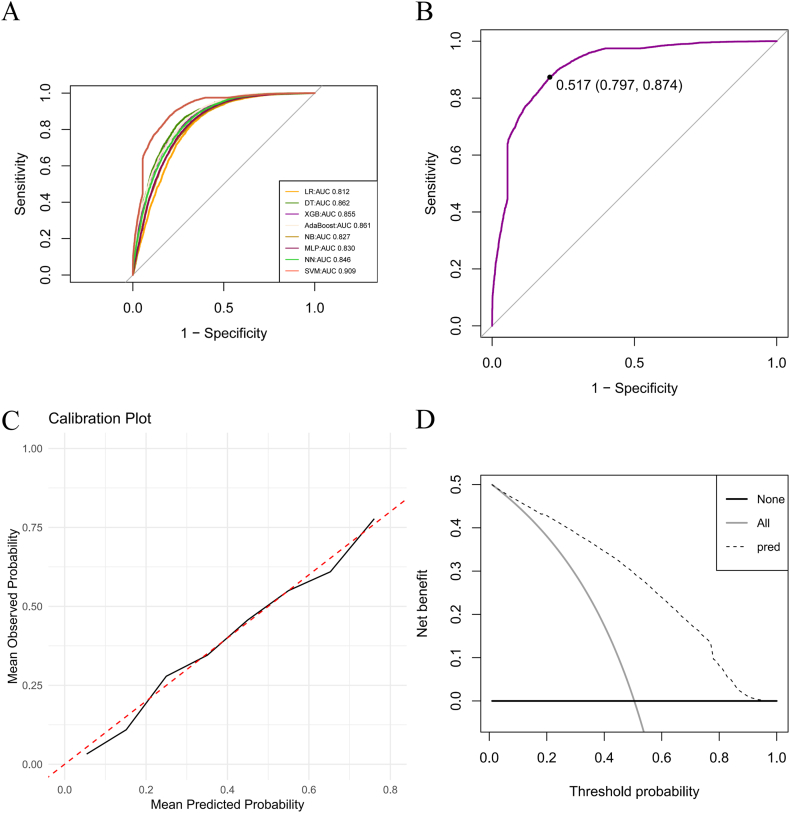
Plot (A) **Receiver operating characteristic (ROC) curves of different machine learning models** The curves show the predictive performance of logistic regression (LR; AUC = 0.812), decision tree (DT; AUC = 0.862), Extreme Gradient Boosting (XGBoost; AUC = 0.855), Adaptive Boosting (AdaBoost; AUC = 0.861), Naive Bayes (NB; AUC = 0.827), multilayer perceptron (MLP; AUC = 0.830), neural network (NN; AUC = 0.846), and support vector machine (SVM; AUC = 0.909), with sensitivity versus 1-specificity. Different colors represent different machine learning models. Plot (B) **Youden's index analysis of the classifier performance** The ROC curve (SVM) demonstrates the trade-off between sensitivity (0.797) and specificity (0.874), with the optimal cutoff threshold of 0.517 identified by Youden's index. Plot (C) **Calibration curve** The curve illustrates the relationship between predicted values from the model and actual observed outcomes. Plot (D) **Decision curves** The decision curves display the net benefits of using the predictive model at various threshold probabilities. (For interpretation of the references to color in this figure legend, the reader is referred to the Web version of this article.)

**Supplementary Fig. 2:**
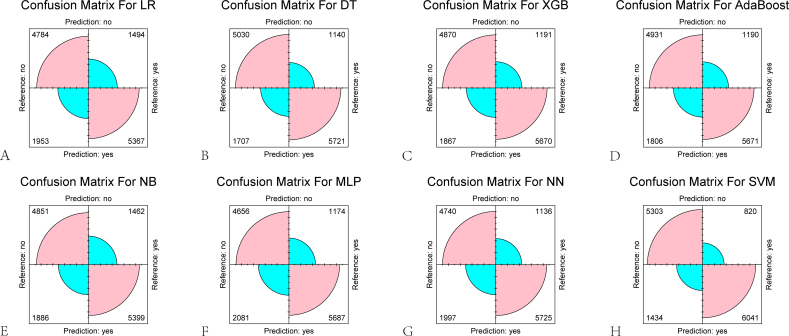
**Confusion matrices of different machine learning models** The matrices show the prediction performance of (A) Logistic Regression (LR), (B) Decision Tree (DT), (C) Extreme Gradient Boosting (XGBoost), (D) Adaptive Boosting (AdaBoost), (E) Naive Bayes (NB), (F) Multilayer Perceptron (MLP), (G) Neural Network (NN), and (H) Support Vector Machine (SVM) against reference labels.

After the preliminary selection of the eight important variables, eight different machine learning models-SVM, NN, NB, XGBoost, MLP, GLM, AdaBoost, and DT-were developed. The evaluation metrics for each model were computed, and confusion matrices were generated (Supplementary Fig. 2). A comparison of the various parameters is provided in Table 2, revealing that the SVM model exhibited the best AUC performance at 0.909 (Fig. 2A) and an average accuracy rate of 83.4 % (82.8 %–84.1 %), indicating that it performs well in identifying CHD. When the validation dataset was input into the SVM model, the AUC remained high at 0.835, suggesting that even with unfamiliar data, the SVM model is capable of maintaining strong recognition performance, further confirming its stability and generalizability.

**Table 2:** Comparison of machine learning performance of different models.

Models	Accuracy(95 % CI)	Precision	Sensitivity	Specificity	Recall	F1	AUC
LR	0.747(0.739,0.754)	0.733	0.782	0.710	0.782	0.757	0.812
DT	0.791(0.784,0.797)	0.770	0.834	0.747	0.834	0.801	0.862
XGBoost	0.775(0.768,0.782)	0.752	0.826	0.723	0.826	0.788	0.855
AdaBoost	0.780(0.773,0.787)	0.758	0.827	0.732	0.827	0.791	0.861
NB	0.754(0.747,0.761)	0.741	0.787	0.720	0.787	0.763	0.827
MLP	0.761(0.753,0.768)	0.732	0.829	0.691	0.829	0.777	0.830
NN	0.770(0.762,0.777)	0.741	0.834	0.704	0.834	0.785	0.846
SVM	0.834(0.828,0.841)	0.808	0.880	0.787	0.880	0.843	0.909

#### 3.1.4 Evaluation and optimization of the best model

The optimal machine learning model for predicting CHD was identified as the SVM. In machine learning models, a higher Youden's J statistic indicates a stronger overall classification capability [39]. The Youden's J statistic for the SVM model was found to be 0.517, with the best cut-off value achieving a sensitivity of 79.7 % and specificity of 87.4 % (Fig. 2B). These results demonstrate the model's solid performance in distinguishing positive cases (those with CHD) from negative cases (those without CHD). Subsequently, hyperparameter tuning was performed using a grid search algorithm, with the sigma parameter set to (0.01, 0.02, 0.05, 0.1) and the C parameter set to (1, 2, 3, 4, 5). This parametric setup systematically evaluated the performance of the SVM model under different combinations for the classification task, ultimately selecting the optimal parameter combination to enhance model accuracy. Following the optimization, evaluation on the test dataset yielded an AUC of 0.909, while the AUC for the validation dataset improved to 0.855. This result not only reflects a significant enhancement in the model's accuracy and generalizability but also indicates that the optimized model is more effective at differentiating between CHD patients and non-patients.

To further evaluate the performance of the optimal SVM model, we generated a calibration curve, as shown in Fig. 2C. The curve demonstrates a good fit with the diagonal line (dashed), indicating a high consistency between the model's predictions and the actual outcomes. Additionally, we plotted a decision curve to assess the net benefit of the model. As illustrated in Fig. 2D, the prediction curve is above both the All and None lines across most of the range (0, 1), suggesting that the model holds practical utility in clinical predictions.

#### 3.1.5 SHAP interpretability analysis

**Fig. 3:**
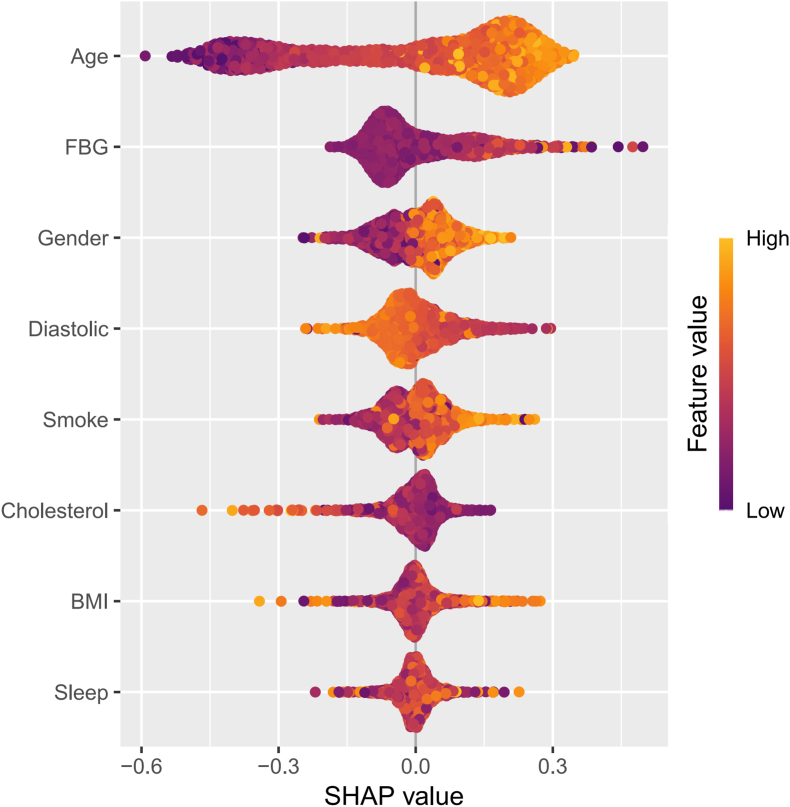
**SHAP Interpretable** This figure displays the SHAP values for the features used in the predictive model. On the left side, features are ranked in order of importance from top to bottom based on their SHAP values. The right side of the plot employs a color gradient, where yellow indicates higher parameter values and purple signifies lower parameter values. This visualization helps to elucidate each feature's contribution to the model's predictions and highlights the variables that have the most significant impact on the outcome. Caution: The negative SHAP values for DBP reflect reverse causation—low DBP is driven by advanced CHD or its treatment (see Discussion and Supplementary Table 2) and should not be interpreted as a protective risk factor. (For interpretation of the references to color in this figure legend, the reader is referred to the Web version of this article.)

**Supplementary Fig. 3:**
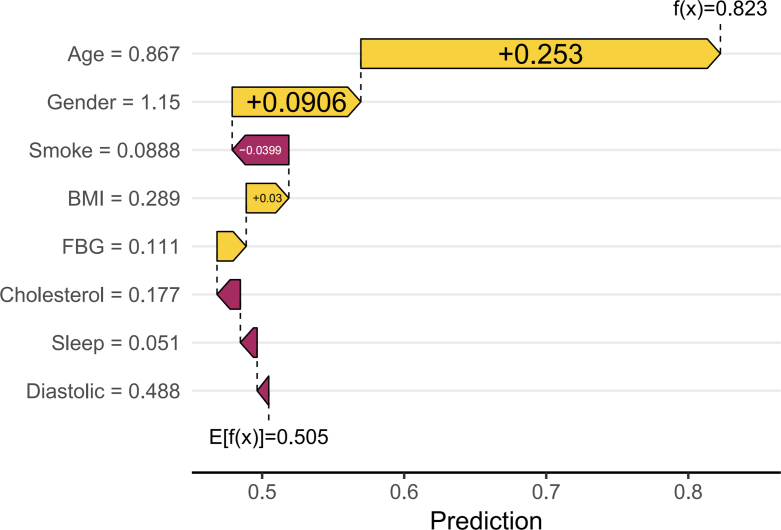
**Waterfall diagram** This waterfall diagram illustrates the cumulative effects of different factors on the predicted outcome. The diagram starts with the baseline measure and sequentially incorporates each variable's effect, allowing for a clear visualization of how various factors influence the overall prediction. This single-subject explanation is provided only to demonstrate feature weights and should not be interpreted as population-level effect directions.

SHAP was employed to visually depict the impact of significant variables on CHD. Fig. 3 illustrates the influence of each variable on the detection dataset's ability to identify CHD within the SVM model. On the left panel of the figure, the variables are ranked in order of importance based on their SHAP values, from top to bottom. The right panlee of the figure shows that yellow indicates higher parameter values, while purple represents lower values. Furthermore, a waterfall plot (Supplementary Fig. 3) is presented to exemplify a single prediction. In the plot, the estimated model value is 0.823, while the predicted value for the sample is 0.505. The variables contributing most significantly to the model are age, sex, smoking status, BMI, and fasting blood glucose levels.

### 3.2 Mendelian randomization component

#### 3.2.1 Instrumental variable selection for mendelian randomization

Following the identification of eight significant factors affecting CHD through machine learning, SHAP interpretability analysis was used to rank these factors by importance. Due to the consensus that age and sex are critical factors influencing CHD and the difficulties in obtaining relevant SNPs for these variables from the GWAS data, the remaining six factors were selected for MR analysis. The GWAS ID for the exposure variables, along with the number of SNPs excluded during the selection process due to duplicate naming and palindrome SNPs with intermediate alleles, is summarized in Table 3. The outcome variable was standardized as CHD, with the GWAS ID designated as ukb-d-I9_CHD.

**Table 3:** Statistical table of exposure variables.

Variables	GWAS ID	Unit of Measurement	Exclusion of SNPs	Final SNPs	Heterogeneity P-value	Horizontal polytropic p-value
FBG	ebi-a-GCST000568	Per 1 mmol/L	0	14	0.066	0.404
Diastolic	ebi-a-GCST90000059	Per 10 mmHg	4	199	<0.001	0.818
Insomnia	ukb-b-3957	Odds (Ever vs. Never)	3	39	0.007	0.078
BMI	ukb-b-19953	Per 1 SD (kg/m^2^)	17	441	<0.001	0.083
TC	ieu-a-301	Per 1 SD (mmol/L)	2	86	<0.001	0.155
Smoke	ebi-a-GCST90029014	Odds (Ever vs. Never)	9	120	0.038	0.333

#### 3.2.2 Results and testing of mendelian randomization

**Fig. 4:**
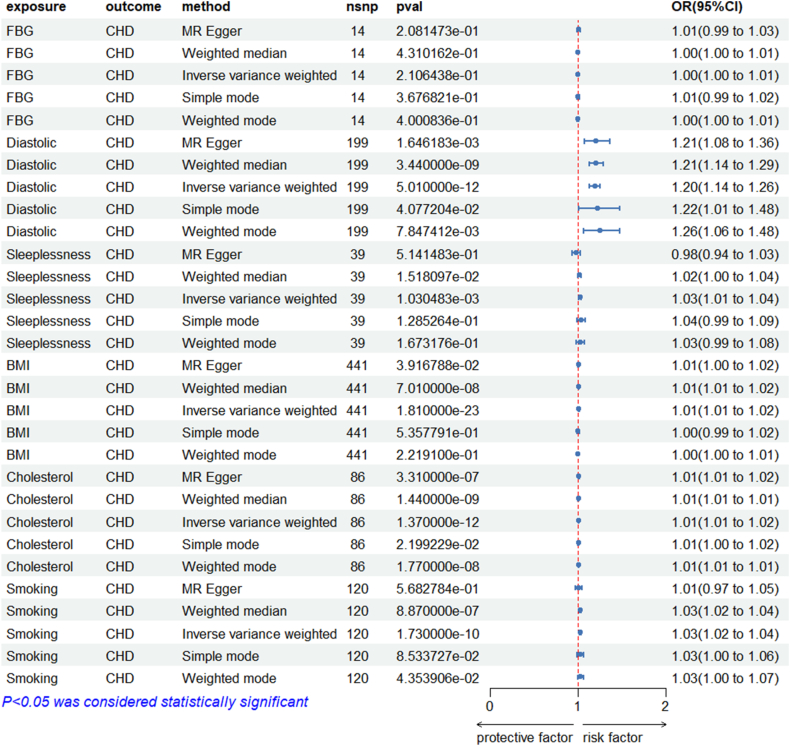
**Summary forest plot of the results of Mendelian randomization** The analysis was conducted using multiple methods, including MR Egger, weighted median, inverse variance weighted, simple mode, and weighted mode. The p-value threshold for statistical significance was set at P < 0.05. The forest plot at the bottom indicates the direction of the effect, with protective factors on the left and risk factors on the right.

The results of the five Mendelian Randomization analyses for fasting blood glucose indicated *P* > 0.05 (Fig. 4), suggesting a lack of statistical significance. Furthermore, both the heterogeneity test and the horizontal pleiotropy test yielded results of *P* > 0.05 (Table 3), indicating that there is no evidence of heterogeneity or horizontal pleiotropy in the analysis.

**Supplementary Fig. 4:**
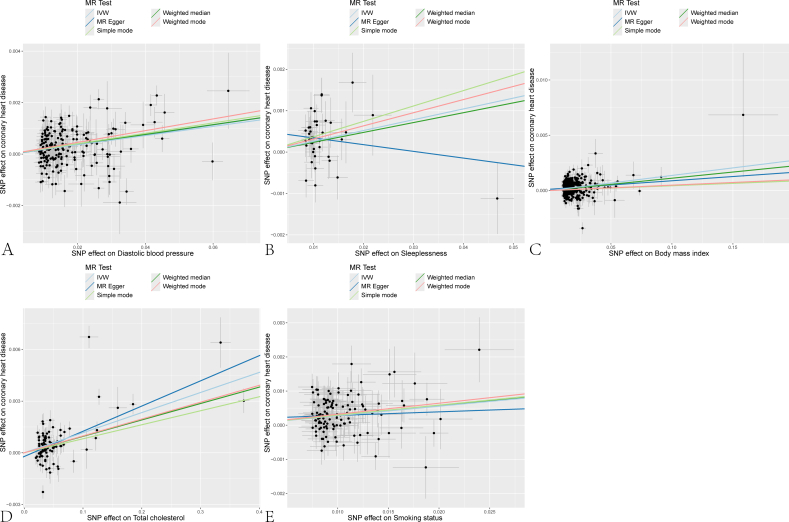
**Summary scatter plots of the results of Mendelian randomization** Plot (A) shows the association between diastolic blood pressure and coronary heart disease; Plot (B) shows the association between insomnia and coronary heart disease; Plot (C) shows the association between BMI and coronary heart disease; Plot (D) shows the association between total cholesterol and coronary heart disease; and Plot (E) shows the association between smoking and coronary heart disease.

**Supplementary Fig. 5:**
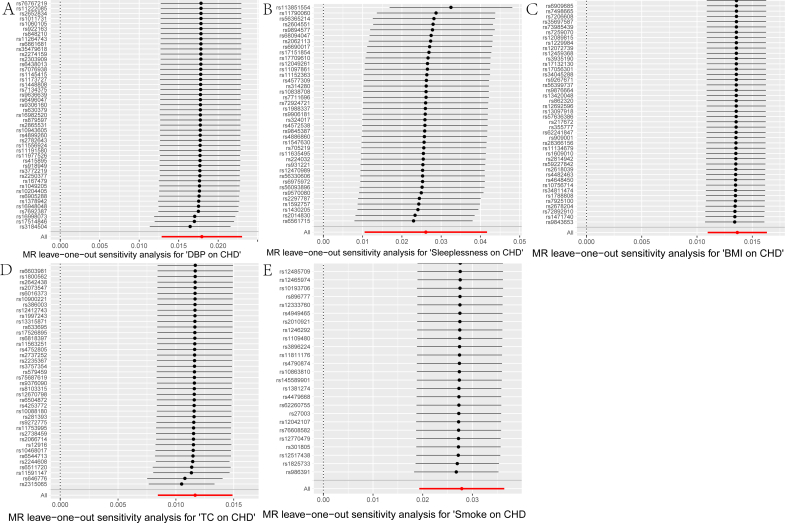
**Summary Leave-one-out plots of the results of Mendelian randomization** Plot (A) shows the association between diastolic blood pressure and coronary heart disease; Plot (B) shows the association between insomnia and coronary heart disease; Plot (C) shows the association between BMI and coronary heart disease; Plot (D) shows the association between total cholesterol and coronary heart disease; and Plot (E) shows the association between smoking and coronary heart disease.

**Supplementary Fig. 6:**
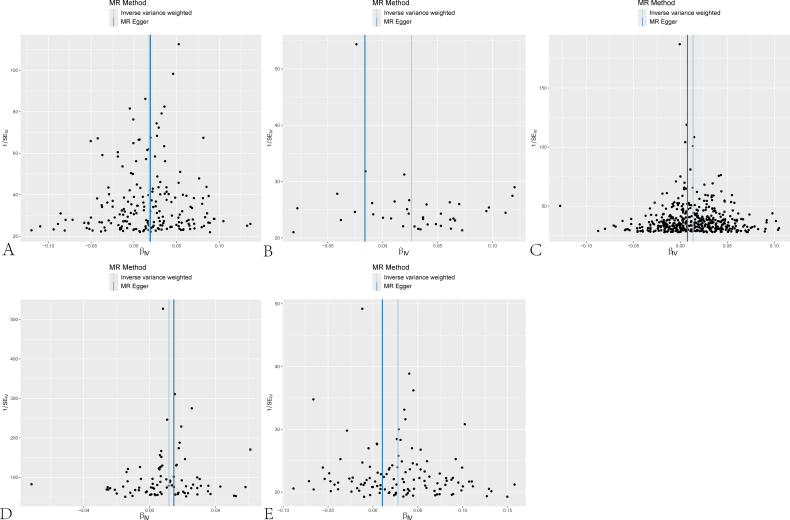
**Summary funnel plots of the results of Mendelian randomization** Plot (A) shows the association between diastolic blood pressure and coronary heart disease; Plot (B) shows the association between insomnia and coronary heart disease; Plot (C) shows the association between BMI and coronary heart disease; Plot (D) shows the association between total cholesterol and coronary heart disease; and Plot (E) shows the association between smoking and coronary heart disease.

The results of the five Mendelian Randomization analyses for diastolic blood pressure revealed *P* < 0.05 (Fig. 4), indicating statistical significance. The heterogeneity test yielded *P* < 0.001, suggesting the presence of certain heterogeneity, which prompts us to focus on the random IVW results. The horizontal pleiotropy test indicated *P* > 0.05, demonstrating no evidence of horizontal pleiotropy (Table 3). The scatter plot (Supplementary Fig. 4A) suggests a consistent directional effect among the five methods, indicating that an increase in diastolic blood pressure is associated with an elevated risk of developing CHD. The leave-one-out analysis results (Supplementary Fig. 5A) show all error bars on one side of zero, while the funnel plot (Supplementary Fig. 6A) reflects a relatively even distribution of points around the IVW method, confirming the robustness of the study. These results suggest that diastolic blood pressure exerts a causal effect on CHD, with an odds ratio (OR) of 1.02 (1.01, 1.02).

In the five MR analyses for insomnia, the *P*-values for the IVW and WME methods were less than 0.05, while the *P*-values for the remaining three methods were greater than 0.05, indicating partial statistical significance (Fig. 4). The heterogeneity test yielded a *P* < 0.05, suggesting the presence of some heterogeneity, which indicates a need to focus on the random IVW results. The horizontal pleiotropy test indicated *P* > 0.05, demonstrating no evidence of horizontal pleiotropy (Table 3). In the scatter plot (Supplementary Fig. 4B), the direction of the MR-Egger method was opposite to that of the other four methods; however, it did not achieve statistical significance. In contrast, both the IVW and WME methods showed consistent directionality, suggesting that as the rate of insomnia increases, the risk of developing CHD also rises. The leave-one-out analysis (Supplementary Fig. 5B) revealed that all error bars were confined to one side of zero, and the funnel plot (Supplementary Fig. 6B) illustrated a relatively even distribution of points around the IVW method, reinforcing the robustness of the findings. The results indicate that insomnia has a causal effect on CHD, with OR of 1.03 (1.01, 1.04).

In the analyses for BMI, the *P*-values for MR-Egger, IVW, and WME were less than 0.05, while the *P*-values for the remaining two methods were greater than 0.05, indicating partial statistical significance (Fig. 4). The heterogeneity test yielded a *P* < 0.001, suggesting the presence of significant heterogeneity, which underscores the importance of focusing on the random IVW results. The horizontal pleiotropy test indicated *P* > 0.05, demonstrating no evidence of horizontal pleiotropy (Table 3). In the scatter plot (Supplementary Fig. 4C), the directions of the MR-Egger, IVW, and WME methods were consistent, indicating that as BMI increases, the risk of developing CHD also rises. The leave-one-out analysis (Supplementary Fig. 5C) showed that all error bars were on one side of zero, and the funnel plot (Supplementary Fig. 6C) reflected a relatively even distribution of points around the IVW method, further confirming the robustness of the findings. The results suggest that an increase in BMI has a causal effect on CHD, with an OR of 1.01 (1.01, 1.02).

The results of the five Mendelian Randomization analyses for TC indicated *P*-values less than 0.05, establishing statistical significance (Fig. 4). The heterogeneity test yielded a *P* < 0.001, suggesting significant heterogeneity, which highlights the need to focus on the random IVW results. The horizontal pleiotropy test returned *P* > 0.05, indicating no evidence of horizontal pleiotropy (Table 3). The scatter plot (Supplementary Fig. 4D) reveals consistent directional effects across all five methods, suggesting that an increase in TC is associated with a heightened risk of developing CHD. In the leave-one-out analysis (Supplementary Fig. 5D), all error bars were located on one side of zero, and the funnel plot (Supplementary Fig. 6D) displayed a relatively even distribution of points around the IVW method, further confirming the robustness of the findings. The results indicate that an increase in total cholesterol has a causal effect on CHD, with an OR of 1.20 (1.14, 1.26).

In the MR analyses for smoking, the *P*-values for IVW, WME, and the weighted model were all less than 0.05, indicating statistical significance. In contrast, the *P*-values for the remaining two methods were greater than 0.05, suggesting partial statistical significance (Fig. 4). The heterogeneity test yielded a *P* < 0.05, indicating the presence of significant heterogeneity, which emphasizes the importance of focusing on the random IVW results. The horizontal pleiotropy test indicated *P* > 0.05, demonstrating no evidence of horizontal pleiotropy (Table 3). The scatter plot (Supplementary Fig. 4E) illustrates consistent directional effects across all five methods, indicating that an increase in smoking is associated with an elevated risk of developing CHD. The leave-one-out analysis (Supplementary Fig. 5E) showed that all error bars were positioned on one side of zero, and the funnel plot (Supplementary Fig. 6E) demonstrated a relatively even distribution of points around the IVW method, reinforcing the robustness of the findings. The results suggest that increased smoking is causally linked to CHD, with an OR of 1.03 (1.02, 1.04).

## 4 Discussion

### 4.1 Key findings

This study employed eight common machine learning methods, including SVM, NN, NB, XGBoost, MLP, GLM, AdaBoost, and DT, to predict CHD based on lifestyle-related variables. Among them, SVM showed the best performance among the eight machine learning models, achieving an AUC of 0.909 and an accuracy of 83.4 %. The results of MR analyses demonstrated positive associations for five variables: diastolic blood pressure, insomnia, BMI, TC, and smoking. These findings suggest that as the parameters of these five variables increase, the risk developing CHD also rises, indicating that these variables significantly influence the occurrence and progression of the disease. Notably, fasting glucose showed predictive but not causal association (P = 0.21), suggesting its role as a biomarker rather than therapeutic target - a critical distinction enabled by our dual-method design.

### 4.2 Comparative performance analysis and innovation

Numerous studies have previously attempted to establish machine learning models for predicting CHD. Liu and Zengjing developed a CHD risk prediction model for patients with human immunodeficiency virus using seven machine learning techniques and electronic health record data. Their results indicated that the Light Gradient Boosting Machine (LightGBM) model exhibited superior overall performance, showing enhanced reliability in assessing risk prediction factors for CHD [40]. In another study, Ma and Cai-Yi analyzed the risk of CHD among more than 300,000 diabetic patients in Southwest China using three traditional machine learning models. The random forest model produced an AUC of 0.701 on the test samples, providing personalized guidance for early CHD warning in the diabetic population [41]. Similarly, Xu and Hu utilized machine learning algorithms to predict CHD among diabetic patients, drawing on data from the General Hospital of the PLA. Their study employed five different machine learning algorithms, finding that the established XGBoost model demonstrated significant predictive value for elderly diabetic patients with CHD, achieving an AUC of 0.880 [42]. Compared to previous studies, this research. Unlike previous studies limited to either prediction or correlation analysis, our research advances CHD risk assessment through three key innovations: (1) systematic comparison of eight machine learning algorithms identifying SVM as optimal (AUC = 0.909, accuracy = 83.4 %) using readily obtainable lifestyle variables; (2) integration of MR to distinguish causal risks (smoking, sleep duration) from mere biomarkers (fasting glucose); and (3) development of a clinically actionable tool requiring only non-invasive measurements, bridging a critical gap between risk prediction and causal prevention strategies.

### 4.3 Model features and evaluation

In this study, we selected demographic data and 16 lifestyle-related factors from the NHANES database as the feature variables. These included age, sex, education level, race, marital status, physical activity, sleep status, smoking, alcohol consumption, BMI, poverty index, fasting blood glucose, systolic blood pressure, diastolic blood pressure, energy intake, protein intake, carbohydrate intake, total sugars, dietary fiber, TC, and total fats, totaling 21 variables. In contrast to most previous studies [[40], [41], [42]], which have predominantly considered biochemical indicators as key variables in CHD prediction models, our model focuses on lifestyle-related indicators. These indicators are generally easier to obtain and less invasive, with only fasting blood glucose being a minimally invasive measurement that can be self-collected at home. Therefore, the models we established operate independently of biochemical indicators. Once high-risk individuals are flagged, their modifiable lifestyle targets (sleep, physical activity, diet, alcohol, smoking) can be delivered to patients and clinicians through mobile health (mHealth) apps and telemedicine portals, tools that have already improved heart-rate variability in high-risk diabetics and are poised to expand scalable, guideline-aligned CHD prevention [43,44]. Our goal was to develop a practical, convenient, and highly acceptable screening model, which can facilitate early identification of individuals at risk of CHD based on easily accessible lifestyle-related factors.

As is shown in Fig. 4, the relative importance of various variables identified in the final selection, with age, gender, fasting glucose, diastolic blood pressure, smoking status, TC, BMI, and sleep quality being the most significant factors influencing model design. Given the inherent complexity of machine learning methods and the challenges in intuitively presenting results, we applied SHAP values for interpretability within the SVM model to achieve optimal variable representation and clarity. A positive SHAP value indicates that the associated feature contributes to a higher risk of CHD, whereas a negative SHAP value indicates a lower risk.

The interpretation of SHAP in conjunction with the causal validation through MR reveals results that align closely with earlier studies on coronary heart disease risk factors. Data from 102 prospective studies indicate that for populations without a history of diabetes, fasting glucose levels or impaired fasting glucose do not significantly improve the predictive metrics for vascular diseases when considered alongside several conventional risk factors [45]. A recent meta-analysis assessing various hypertension diagnostic guidelines found that isolated diastolic hypertension is associated with increased cardiovascular risk, and higher diastolic blood pressure thresholds correlate with elevated cardiovascular risk [46]. Although the ML model correctly reproduces the empirical observation that lower DBP is associated with higher contemporaneous probability of CHD (Fig. 3), this association is unlikely to be causal. Because the model was trained on contemporaneous measurements, the captured associations may reflect consequences of already-established disease or its treatment rather than aetiological risk factors. Supplementary Table 2 shows that participants with manifest CHD were more frequently prescribed antihypertensive medications and had lower cardiac output, both of which reduce measured DBP. Consequently, low DBP should be interpreted as a marker of advanced disease or aggressive treatment rather than a causal risk factor for CHD. We attempted prospective validation using the NHANES 2013–2018 linked mortality files; however, the follow-up duration is short and cardiovascular deaths are too few to meet the minimum event-per-variable requirement of Cox regression. Notably, prior NHANES analyses reported a sharp rise in cardiovascular mortality when DBP exceeded 75 mmHg, a direction consistent with our MR estimates and further supporting a positive causal effect of long-term elevated DBP on CHD [47]. Hypertension is widely considered as an independent risk factor for CHD; however, initial discussions often prioritize systolic blood pressure, leading to an underappreciation of diastolic pressure. Our study demonstrates that diastolic pressure plays an indispensable role in the risk associated with coronary heart disease, suggesting that healthcare professionals and the public should equally weigh diastolic pressure alongside systolic pressure when evaluating cardiovascular health. Existing data indicate that insomnia is linked with an increased risk of hypertension, coronary heart disease, and recurrent acute coronary syndrome [48]. Due to societal pressures and health-related issues, insomnia has become a pervasive sleep disorder, significantly impacting the daily lives and mental health of many individuals. Our study further validates the significant role of insomnia in the risk of CHD, indicating that the cardiovascular health risks among individuals with insomnia may be more severe than those in the general population. We emphasize the importance of sleep quality and encourage the public to develop good sleep habits, which could potentially incidence CHD Regarding the well-known cardiovascular risk factor, BMI, a prospective cohort study from the China Biobank indicates that participants who are overweight have a 41 % higher risk of developing CHD compared to those with BMI [49]. With the advancement technology and the rise a smart society, increasing of individuals are opting for modes of, resulting in a general increase in BMI. To mitigate the risk of CHD, recommend that individuals engage in exercise, their BMI, and maintain a healthy. Elevated levels of TC have long been recognized as one of the primary causes of atherosclerosis and cardiovascular diseases. We emphasize that the “cholesterol” entry in Table 1 quantifies dietary cholesterol intake, whereas the MR exposure is based on serum TC. The inverse cross-sectional association between dietary cholesterol and prevalent CHD (Table 1) is therefore not contradictory to the positive genetically-predicted effect of serum cholesterol on incident CHD (Fig. 4). The observed inverse association likely reflects reverse causation: individuals with diagnosed CHD frequently reduce consumption of high-cholesterol foods, attenuating their apparent intake. Circulating cholesterol, in contrast, captures systemic lipid metabolism that causally elevates CHD risk. This distinction underscores the limitation of interpreting dietary variables as proxies for biological exposure and reinforces the need for MR designs that leverage serum lipid biomarkers. Our MR analysis of TC implicates genetically elevated TC as a causal CHD driver; this aligns with evidence that saturated fat and added sugar—rather than dietary cholesterol itself—are the main dietary determinants of TC. Reducing animal-source foods rich in these fats and sugars (e.g., fatty red meat, full-fat dairy, sugar-sweetened items) would therefore be expected to lower serum TC and consequent CHD risk. Smoking has long been recognized as an independent risk factor for cardiovascular diseases, and our study similarly highlights its significant impact on the risk of CHD. Research indicates that the risk of cardiovascular events decreases significantly within five years after quitting smoking, and this risk can diminish to levels comparable to never smokers over several decades [50]. This finding suggests that it is never too late to take action to quit smoking, regardless of when one begins the cessation process. It is crucial for individuals to recognize the harmful effects of smoking on health; distancing oneself from cigarettes can effectively reduce the risk of CHD. By quitting smoking, individuals not only improve their own health status but also contribute to creating a healthier environment for those around them.

### 4.4 Limitations and future directions

Several limitations are present in our study. First, the diagnosis of CHD was defined based on self-reported data from interview questionnaires in the NHANES database, which may introduce information bias due to cognitive deficits or recall bias. Any inaccuracies in CHD classification could, to some extent, affect the accuracy of machine learning models in identifying CHD. Second, during the inclusion of overall data for machine learning model analysis, there were still some missing metrics for certain participants. Although we employed rigorous statistical methods for imputation, some residual bias may persist. Furthermore, as our study is based on the NHANES database and the GWAS gene repository, the results may only be applicable to the U.S. population and may not be directly generalizable to other global populations. Future research should focus on validating our model across diverse populations, such as those from UK and Asian biobanks, while also incorporating clinical diagnoses, including data from electronic health records, to mitigate self-report bias. Additionally, the use of advanced methodologies like federated learning, deep learning architectures, and multi-omics integration, such as proteomics, has the potential to enhance the model's robustness and biological interpretability. Moreover, implementation studies are crucial to assess the real-world applicability, cost-effectiveness, and development of clinician-friendly interfaces, which are essential for the scalable adoption of the model in preventive healthcare.

## 5 Conclusion

While previous researchers have developed predictive models for CHD, most of these models have incorporated laboratory metrics from hospital settings and utilized traditional statistical methods. With the continuous advancement and refinement of machine learning in recent years, its applications in healthcare have become increasingly prevalent. This study demonstrates the potential of using lifestyle-related data to predict CHD through machine learning methodologies. We utilized data from 29,400 respondents from the NHANES, incorporating 21 lifestyle variables and applying eight common machine learning models for analysis. Following model training, we evaluated and compared the models using the AUC. The performance ranking of the eight machine learning models was SVM > DT > AdaBoost > XGBoost > NN > MLP > NB > GLM, with the SVM model achieving an accuracy of 83.4 % and an AUC of 0.909. Subsequently, SHAP interpretability and Mendelian randomization validation were performed, and our cumulative results indicate that the SVM model can better predict CHD using lifestyle-related indicators. In daily life, individuals can reduce their risk of developing CHD by minimizing cholesterol intake, paying attention to changes in diastolic blood pressure, managing sleep duration appropriately, maintaining BMI within a normal range, and avoiding smoking or quitting as early as possible. To summarize, by synergizing machine learning and MR, we developed and causally validated a practical CHD prediction tool. Our findings shift prevention paradigms from biomarker-centric approaches to modifiable lifestyle factors, offering a scalable strategy to reduce the global CHD burden.

## CRediT authorship contribution statement

**Yang yang Cui:** Writing – original draft, Methodology, Investigation, Formal analysis. **Yonghong Zhang:** Writing – review & editing, Software, Methodology. **Lang Zeng:** Writing – review & editing, Methodology, Investigation. **Shikang Li:** Validation, Methodology, Investigation. **Xue Mei:** Investigation. **Xiangmei Yang:** Writing – review & editing, Investigation. **Peng Zhou:** Investigation. **Lijuan Xiong:** Investigation. **Yijuan Huang:** Software, Resources. **Jing Luo:** Investigation. **Fenglin Wu:** Supervision, Conceptualization. **Rongchuan Yue:** Writing – review & editing, Writing – original draft, Methodology, Funding acquisition, Conceptualization.

## Funding

This study was supported by the Nanchong science and technology plan project (23JCYJPT0059); Research and development program of North Sichuan Medical College (CBY23-TD01); Research and Development Plan project of Clinical School and Affiliated Hospital of North Sichuan Medical University(2024PTZK007); Scientific Research Program of Sichuan Medical Association(2024TG38) and Funding from the Scientific Startup Foundation for Doctors of North Sichuan Medical College to Xue Mei (CBY23-QDA11).

## References

[1] de Oliveira Laterza Ribeiro M., Correia V.M., Herling de Oliveira L.L., Soares P.R., Scudeler T.L. (2023). Evolving diagnostic and management advances in coronary heart disease. Life.

[2] Tang M.M., Zhao S.T., Li R.Q., Hou W. (2023). Therapeutic mechanisms of ginseng in coronary heart disease. Front. Pharmacol..

[3] Wang N., Sun Y., Zhang H. (2021). Long-term night shift work is associated with the risk of atrial fibrillation and coronary heart disease. Eur. Heart J..

[4] Ferreira-González I. (2014). The epidemiology of coronary heart disease. Rev. Esp. Cardiol..

[5] Al-Shaar Laila (2020). Red meat intake and risk of coronary heart disease among US men: prospective cohort study. BMJ Clin. Res..

[6] Kononenko I. (2001). Machine learning for medical diagnosis: history, state of the art and perspective. Artif. Intell. Med..

[7] Qin Yifan (2022). Machine learning models for data-driven prediction of diabetes by lifestyle type. Int. J. Environ. Res. Publ. Health.

[8] Hassan Ch Anwar Ul (2022). Effectively predicting the presence of coronary heart disease using machine learning classifiers. Sensors (Basel, Switzerland).

[9] Yilmaz A., Hayıroğlu M.İ., Salturk S. (2023). Machine learning approach on high risk treadmill exercise test to predict obstructive coronary artery disease by using P, QRS, and T waves' features. Curr. Probl. Cardiol..

[10] Cicek V., Orhan A.L., Saylik F. (2025). Predicting short-term mortality in patients with acute pulmonary embolism with deep learning. Circ. J..

[11] Zhang Liying (2022). Nonlaboratory-based risk assessment model for coronary heart disease screening: model development and validation. Int. J. Med. Inf..

[12] Naderian Mohammadreza (2024). Development and evaluation of a comprehensive prediction model for incident coronary heart disease using genetic, social, and lifestyle-psychological factors: a prospective analysis of the UK biobank. Annals of internal medicine.

[13] Paulose-Ram Ryne (2021). The National health and Nutrition Examination Survey (NHANES), 2021-2022: adapting data collection in a COVID-19 environment. American journal of public health.

[14] Li Xi (2023). Development of an interpretable machine learning model associated with heavy metals' exposure to identify coronary heart disease among US adults via SHAP: findings of the US NHANES from 2003 to 2018. Chemosphere.

[15] Lloyd-Jones Donald M. (2022). Life's essential 8: updating and enhancing the American heart association's construct of cardiovascular health: a presidential advisory from the American heart Association. Circulation.

[16] Daberdaku Sebastian (2020). A combined interpolation and weighted K-Nearest neighbours approach for the imputation of longitudinal ICU laboratory data. Journal of healthcare informatics research.

[17] Uddin Md Jamal, Fan Jitang (2024). Interpretable machine learning framework to predict the glass transition temperature of polymers. Polymers.

[18] Llanos Fernando (2023). The relationship between sentence intelligibility, band importance, and signal covariance. JASA express letters.

[19] Poldrack Russell A. (2020). Establishment of best practices for evidence for prediction: a review. JAMA Psychiatry.

[20] Albogamy Fahad R. (2022). Decision support System for predicting survivability of hepatitis patients. Front. Public Health.

[21] Vandewiele Gilles (2021). Overly optimistic prediction results on imbalanced data: a case study of flaws and benefits when applying over-sampling. Artif. Intell. Med..

[22] Budhathoki Nirajan (7 Dec. 2023). Predicting asthma using imbalanced data modeling techniques: evidence from 2019 Michigan BRFSS data. PLoS One.

[23] Zhang Zhaohui (2023). Identification of potential feature genes in non-alcoholic fatty liver disease using bioinformatics analysis and machine learning strategies. Comput. Biol. Med..

[24] Ding Chen (2022). Quantum-Inspired support vector machine. IEEE Transact. Neural Networks Learn. Syst..

[25] Esteva Andre (2017). Dermatologist-level classification of skin cancer with deep neural networks. Nature.

[26] Golpour Parastoo (2020). Comparison of support vector machine, Naïve bayes and logistic regression for assessing the necessity for coronary angiography. Int. J. Environ. Res. Publ. Health.

[27] Li Qingqing (2022). XGBoost-based and tumor-immune characterized gene signature for the prediction of metastatic status in breast cancer. J. Transl. Med..

[28] Zhang Zheyu (26 Oct. 2022). Multilayer perceptron-based prediction of stroke mimics in prehospital triage. Sci. Rep..

[29] McCabe Connor J. (2022). Interpreting interaction effects in generalized Linear models of nonlinear probabilities and counts. Multivariate Behav. Res..

[30] Hisham B., Hamouda A. (2021). Arabic sign language recognition using Ada-Boosting based on a leap motion controller. Int. j. inf. tecnol..

[31] Zweck Elric (2021). Machine learning identifies clinical parameters to predict mortality in patients undergoing transcatheter mitral valve repair. JACC Cardiovasc. Interv..

[32] Pruessner Jens C. (2003). Two formulas for computation of the area under the curve represent measures of total hormone concentration versus time-dependent change. Psychoneuroendocrinology.

[33] Larsson Susanna C. (2023). Mendelian randomization for cardiovascular diseases: principles and applications. Eur. Heart J..

[34] Davey Smith George, Hemani Gibran (2014). Mendelian randomization: genetic anchors for causal inference in epidemiological studies. Hum. Mol. Genet..

[35] Hartwig Fernando Pires (2016). Two-sample Mendelian randomization: avoiding the downsides of a powerful, widely applicable but potentially fallible technique. Int. J. Epidemiol..

[36] Bowden Jack (2018). Improving the visualization, interpretation and analysis of two-sample summary data Mendelian randomization via the Radial plot and Radial regression. Int. J. Epidemiol..

[37] Li Biyun (2024). The causal relationship between gut microbiota and lymphoma: a two-sample Mendelian randomization study. Front. Immunol..

[38] Gronau Quentin F., Eric-Jan Wagenmakers (2019). Limitations of Bayesian leave-one-out cross-validation for model selection. Computational brain & behavior.

[39] Li Chenxue (2019). Partial Youden index and its inferences. J. Biopharm. Stat..

[40] Liu Zengjing (2024). Predictive model and risk analysis for coronary heart disease in people living with HIV using machine learning. BMC Med. Inf. Decis. Making.

[41] Ma Cai-Yi (2024). Predicting coronary heart disease in Chinese diabetics using machine learning. Comput. Biol. Med..

[42] Xu Hu (2022). Establishment of a diagnostic model of coronary heart disease in elderly patients with diabetes mellitus based on machine learning algorithms. Journal of geriatric cardiology : JGC.

[43] Hayıroğlu M.İ. (2019). Telemedicine: current concepts and future perceptions. Anatol. J. Cardiol..

[44] Hayıroğlu M.İ., Çinier G., Yüksel G. (2021). Effect of a mobile application and smart devices on heart rate variability in diabetic patients with high cardiovascular risk: a sub-study of the LIGHT randomized clinical trial. Kardiol. Pol..

[45] Emerging Risk Factors Collaboration (2010). Diabetes mellitus, fasting blood glucose concentration, and risk of vascular disease: a collaborative meta-analysis of 102 prospective studies. Lancet (London, England).

[46] Agarwal Abhimanyu (2024). Isolated diastolic hypertension and cardiovascular outcomes across different diagnostic guidelines: a systematic review and meta-analysis. The Egyptian heart journal : (EHJ) : official bulletin of the Egyptian Society of Cardiology.

[47] Elfassy T., German C.A., Muntner P. (2023). Blood pressure and cardiovascular disease mortality among US adults: a sex-stratified analysis, 1999-2019. Hypertension.

[48] Javaheri Sogol, Redline Susan (2017). Insomnia and risk of cardiovascular disease. Chest.

[49] Song J.H. (2022). Zhonghua liu xing bing xue za zhi = Zhonghua liuxingbingxue zazhi.

[50] Rigotti Nancy A., McDermott Mary M. (2019). Smoking cessation and cardiovascular disease: it's never too early or too late for action. J. Am. Coll. Cardiol..

