# Supplementary material for: Machine learning and Mendelian randomization identify key lifestyle factors in coronary heart disease: A NHANES-Based study
Source: Int J Cardiol Cardiovasc Risk Prev. 2025 Oct 26;27:200536. doi: 10.1016/j.ijcrp.2025.200536 (PMC12637256; doi:10.1016/j.ijcrp.2025.200536)
Supplement: Multimedia component 1 [file mmc1.docx]

| **Supplementary Table 1. A complete list of SNPs and related information** | | | | | | | | |
| --- | --- | --- | --- | --- | --- | --- | --- | --- |
|  | SNP | effect allele | other allele | freq of effect allele | beta | SE | N | F statistics |
| FBG | rs780093 | C | T | 0.606 | 0.027 | 0.004 | 46186 | 53.248 |
|  | rs560887 | C | T | 0.674 | 0.075 | 0.004 | 46186 | 334.608 |
|  | rs11717195 | C | T | 0.770 | -0.029 | 0.005 | 46186 | 38.070 |
|  | rs2191349 | T | G | 0.467 | 0.030 | 0.004 | 46186 | 69.441 |
|  | rs10276674 | C | T | 0.836 | 0.036 | 0.005 | 46186 | 49.825 |
|  | rs6975024 | C | T | 0.800 | 0.062 | 0.005 | 46186 | 160.093 |
|  | rs11558471 | G | A | 0.748 | -0.027 | 0.004 | 46186 | 45.561 |
|  | rs4506565 | T | A | 0.704 | 0.023 | 0.004 | 46186 | 33.061 |
|  | rs10787312 | A | G | 0.078 | 0.042 | 0.006 | 46186 | 45.888 |
|  | rs7944584 | T | A | 0.712 | -0.025 | 0.004 | 46186 | 37.179 |
|  | rs2524299 | T | A | 0.892 | -0.030 | 0.005 | 46186 | 33.283 |
|  | rs10830963 | G | C | 0.700 | 0.079 | 0.006 | 46186 | 308.184 |
|  | rs12805422 | A | G | 0.500 | -0.023 | 0.004 | 46186 | 38.640 |
|  | rs17390909 | G | C | 0.883 | -0.037 | 0.007 | 46186 | 30.495 |
|  |  |  |  |  |  |  |  |  |
| Diastolic | rs4601530 | T | C | 0.254 | 0.018 | 0.002 | 810865 | 97.883 |
|  | rs1011731 | A | G | 0.568 | -0.014 | 0.002 | 810865 | 80.789 |
|  | rs2250377 | G | A | 0.668 | -0.011 | 0.002 | 810865 | 40.169 |
|  | rs2493292 | T | C | 0.143 | 0.023 | 0.002 | 810865 | 102.065 |
|  | rs848210 | G | A | 0.574 | -0.011 | 0.002 | 810865 | 46.803 |
|  | rs11264743 | T | C | 0.295 | -0.010 | 0.002 | 810865 | 36.463 |
|  | rs12748152 | T | C | 0.081 | 0.016 | 0.003 | 810865 | 32.011 |
|  | rs35479618 | A | G | 0.017 | 0.064 | 0.006 | 810865 | 106.086 |
|  | rs699 | G | A | 0.408 | 0.023 | 0.002 | 810865 | 209.220 |
|  | rs1138293 | T | C | 0.193 | -0.013 | 0.002 | 810865 | 41.336 |
|  | rs17391694 | T | C | 0.134 | -0.019 | 0.002 | 810865 | 70.288 |
|  | rs17030613 | C | A | 0.214 | 0.026 | 0.002 | 810865 | 178.936 |
|  | rs6662118 | G | A | 0.373 | 0.024 | 0.002 | 810865 | 189.834 |
|  | rs17367504 | G | A | 0.160 | -0.045 | 0.002 | 810865 | 444.844 |
|  | rs2782643 | T | C | 0.417 | 0.012 | 0.002 | 810865 | 58.400 |
|  | rs2527614 | G | A | 0.526 | -0.013 | 0.002 | 810865 | 71.646 |
|  | rs1657502 | G | T | 0.382 | 0.017 | 0.002 | 810865 | 107.468 |
|  | rs6714498 | C | T | 0.378 | -0.010 | 0.002 | 810865 | 33.395 |
|  | rs653220 | G | A | 0.239 | -0.012 | 0.002 | 810865 | 40.016 |
|  | rs17020136 | C | T | 0.195 | -0.014 | 0.002 | 810865 | 48.151 |
|  | rs3816183 | C | T | 0.744 | -0.012 | 0.002 | 810865 | 42.903 |
|  | rs33979934 | A | T | 0.267 | -0.012 | 0.002 | 810865 | 43.222 |
|  | rs7578326 | G | A | 0.346 | -0.009 | 0.002 | 810865 | 32.287 |
|  | rs12477314 | T | C | 0.201 | 0.012 | 0.002 | 810865 | 34.855 |
|  | rs2679722 | A | G | 0.633 | 0.010 | 0.002 | 810865 | 34.610 |
|  | rs2381683 | A | G | 0.402 | 0.015 | 0.002 | 810865 | 89.882 |
|  | rs10204405 | A | G | 0.439 | -0.020 | 0.002 | 810865 | 154.569 |
|  | rs17362588 | A | G | 0.088 | 0.031 | 0.003 | 810865 | 127.344 |
|  | rs2053163 | G | A | 0.710 | -0.011 | 0.002 | 810865 | 39.822 |
|  | rs12993599 | A | G | 0.051 | -0.025 | 0.004 | 810865 | 51.310 |
|  | rs11676272 | G | A | 0.479 | -0.017 | 0.002 | 810865 | 107.329 |
|  | rs10495928 | G | A | 0.333 | -0.013 | 0.002 | 810865 | 66.071 |
|  | rs918949 | T | C | 0.602 | -0.015 | 0.002 | 810865 | 89.328 |
|  | rs6795735 | T | C | 0.408 | -0.013 | 0.002 | 810865 | 64.075 |
|  | rs6762208 | A | C | 0.342 | 0.017 | 0.002 | 810865 | 104.177 |
|  | rs2272007 | C | T | 0.834 | -0.031 | 0.002 | 810865 | 205.520 |
|  | rs34173813 | T | C | 0.131 | -0.014 | 0.002 | 810865 | 36.030 |
|  | rs1353776 | G | C | 0.646 | -0.011 | 0.002 | 810865 | 42.236 |
|  | rs16851397 | G | A | 0.048 | 0.039 | 0.004 | 810865 | 113.428 |
|  | rs2606736 | T | C | 0.625 | 0.010 | 0.002 | 810865 | 37.459 |
|  | rs34221642 | C | A | 0.055 | 0.022 | 0.004 | 810865 | 30.722 |
|  | rs6438013 | T | G | 0.319 | -0.012 | 0.002 | 810865 | 48.664 |
|  | rs59508481 | T | C | 0.740 | -0.014 | 0.002 | 810865 | 57.741 |
|  | rs3755652 | T | C | 0.261 | 0.016 | 0.002 | 810865 | 77.486 |
|  | rs1549979 | T | C | 0.623 | 0.011 | 0.002 | 810865 | 46.130 |
|  | rs2303909 | A | G | 0.528 | 0.010 | 0.002 | 810865 | 37.232 |
|  | rs294636 | T | G | 0.817 | -0.015 | 0.002 | 810865 | 56.126 |
|  | rs3772219 | C | A | 0.318 | -0.015 | 0.002 | 810865 | 75.743 |
|  | rs16843947 | G | A | 0.408 | 0.010 | 0.002 | 810865 | 36.395 |
|  | rs448378 | A | G | 0.528 | -0.025 | 0.002 | 810865 | 256.797 |
|  | rs922163 | T | C | 0.641 | -0.010 | 0.002 | 810865 | 41.267 |
|  | rs7698598 | G | A | 0.864 | -0.016 | 0.002 | 810865 | 46.493 |
|  | rs7692387 | A | G | 0.190 | -0.024 | 0.002 | 810865 | 141.848 |
|  | rs10018786 | G | T | 0.102 | 0.020 | 0.003 | 810865 | 57.060 |
|  | rs2320299 | A | G | 0.730 | 0.011 | 0.002 | 810865 | 41.232 |
|  | rs13107325 | T | C | 0.072 | -0.060 | 0.003 | 810865 | 374.333 |
|  | rs4691380 | T | C | 0.324 | -0.011 | 0.002 | 810865 | 42.866 |
|  | rs16998073 | T | A | 0.296 | 0.042 | 0.002 | 810865 | 587.403 |
|  | rs1448808 | G | A | 0.550 | -0.010 | 0.002 | 810865 | 43.192 |
|  | rs7666785 | G | A | 0.599 | -0.009 | 0.002 | 810865 | 30.143 |
|  | rs1582931 | A | G | 0.474 | 0.024 | 0.002 | 810865 | 156.315 |
|  | rs34471628 | G | A | 0.039 | -0.024 | 0.004 | 810865 | 33.774 |
|  | rs832582 | A | G | 0.824 | 0.012 | 0.002 | 810865 | 34.078 |
|  | rs2307111 | C | T | 0.397 | 0.017 | 0.002 | 810865 | 105.375 |
|  | rs13155377 | T | G | 0.660 | 0.012 | 0.002 | 810865 | 51.457 |
|  | rs247008 | G | A | 0.666 | -0.009 | 0.002 | 810865 | 30.853 |
|  | rs1173727 | C | T | 0.596 | 0.029 | 0.002 | 810865 | 335.983 |
|  | rs2549794 | T | C | 0.569 | -0.012 | 0.002 | 810865 | 49.568 |
|  | rs4705752 | G | A | 0.506 | -0.009 | 0.002 | 810865 | 30.172 |
|  | rs6861681 | A | G | 0.311 | -0.017 | 0.002 | 810865 | 99.977 |
|  | rs1799945 | G | C | 0.148 | 0.036 | 0.002 | 810865 | 251.887 |
|  | rs6905288 | A | G | 0.566 | 0.015 | 0.002 | 810865 | 94.169 |
|  | rs4897193 | A | G | 0.512 | 0.022 | 0.002 | 810865 | 192.284 |
|  | rs7756992 | G | A | 0.273 | 0.011 | 0.002 | 810865 | 37.214 |
|  | rs605066 | T | C | 0.586 | -0.011 | 0.002 | 810865 | 38.410 |
|  | rs2328813 | A | C | 0.571 | -0.010 | 0.002 | 810865 | 40.177 |
|  | rs2397060 | C | T | 0.142 | 0.016 | 0.002 | 810865 | 47.868 |
|  | rs10943605 | A | G | 0.486 | 0.015 | 0.002 | 810865 | 96.787 |
|  | rs11153730 | C | T | 0.493 | -0.013 | 0.002 | 810865 | 69.797 |
|  | rs9373985 | G | C | 0.323 | 0.009 | 0.002 | 810865 | 31.545 |
|  | rs339331 | C | T | 0.308 | 0.010 | 0.002 | 810865 | 33.142 |
|  | rs17342717 | T | C | 0.101 | 0.022 | 0.003 | 810865 | 65.839 |
|  | rs3819299 | G | T | 0.061 | 0.020 | 0.004 | 810865 | 30.909 |
|  | rs630379 | C | A | 0.690 | 0.023 | 0.002 | 810865 | 159.697 |
|  | rs881858 | A | G | 0.696 | 0.014 | 0.002 | 810865 | 70.354 |
|  | rs12209452 | G | A | 0.049 | -0.029 | 0.004 | 810865 | 56.315 |
|  | rs11556924 | T | C | 0.385 | -0.016 | 0.002 | 810865 | 94.655 |
|  | rs6969780 | C | G | 0.092 | 0.017 | 0.003 | 810865 | 40.614 |
|  | rs3812316 | G | C | 0.127 | -0.014 | 0.002 | 810865 | 34.198 |
|  | rs1449592 | A | G | 0.516 | -0.010 | 0.002 | 810865 | 43.080 |
|  | rs891511 | A | G | 0.331 | -0.026 | 0.002 | 810865 | 227.637 |
|  | rs10259085 | T | C | 0.432 | 0.010 | 0.002 | 810865 | 36.526 |
|  | rs194520 | G | T | 0.550 | -0.011 | 0.002 | 810865 | 42.157 |
|  | rs11977526 | A | G | 0.399 | 0.010 | 0.002 | 810865 | 38.227 |
|  | rs314370 | C | T | 0.191 | 0.017 | 0.002 | 810865 | 70.716 |
|  | rs144867634 | C | T | 0.025 | -0.029 | 0.005 | 810865 | 34.101 |
|  | rs2469997 | C | G | 0.818 | 0.019 | 0.002 | 810865 | 84.907 |
|  | rs975730 | A | G | 0.353 | -0.009 | 0.002 | 810865 | 32.013 |
|  | rs34591516 | T | C | 0.050 | 0.029 | 0.004 | 810865 | 64.363 |
|  | rs2306899 | T | C | 0.239 | 0.012 | 0.002 | 810865 | 44.428 |
|  | rs7830613 | T | C | 0.315 | 0.014 | 0.002 | 810865 | 63.561 |
|  | rs76767219 | A | C | 0.034 | -0.029 | 0.004 | 810865 | 43.918 |
|  | rs61732533 | A | G | 0.049 | -0.021 | 0.004 | 810865 | 30.336 |
|  | rs4626664 | A | G | 0.154 | 0.013 | 0.002 | 810865 | 38.695 |
|  | rs111245230 | C | T | 0.035 | 0.033 | 0.004 | 810865 | 56.977 |
|  | rs534214 | A | G | 0.547 | 0.011 | 0.002 | 810865 | 44.861 |
|  | rs76452347 | T | C | 0.203 | -0.021 | 0.002 | 810865 | 109.469 |
|  | rs2274159 | G | A | 0.494 | 0.012 | 0.002 | 810865 | 61.097 |
|  | rs7023954 | A | G | 0.423 | 0.010 | 0.002 | 810865 | 36.931 |
|  | rs507666 | A | G | 0.189 | -0.029 | 0.002 | 810865 | 208.509 |
|  | rs3812594 | A | G | 0.273 | 0.010 | 0.002 | 810865 | 30.418 |
|  | rs12773594 | A | T | 0.161 | 0.013 | 0.002 | 810865 | 33.835 |
|  | rs7076938 | T | C | 0.734 | 0.030 | 0.002 | 810865 | 286.368 |
|  | rs60632610 | T | C | 0.143 | 0.015 | 0.002 | 810865 | 43.516 |
|  | rs11191580 | C | T | 0.082 | -0.046 | 0.003 | 810865 | 248.743 |
|  | rs11014166 | T | A | 0.330 | -0.027 | 0.002 | 810865 | 267.060 |
|  | rs1408814 | G | A | 0.580 | -0.009 | 0.002 | 810865 | 31.532 |
|  | rs11555408 | G | T | 0.103 | -0.014 | 0.003 | 810865 | 30.623 |
|  | rs1133400 | G | A | 0.215 | 0.011 | 0.002 | 810865 | 31.313 |
|  | rs7893462 | G | A | 0.528 | 0.010 | 0.002 | 810865 | 42.844 |
|  | rs1530440 | T | C | 0.188 | -0.033 | 0.002 | 810865 | 278.208 |
|  | rs2234962 | C | T | 0.216 | -0.019 | 0.002 | 810865 | 98.004 |
|  | rs16921914 | A | G | 0.286 | 0.011 | 0.002 | 810865 | 33.431 |
|  | rs9344 | A | G | 0.450 | -0.010 | 0.002 | 810865 | 40.779 |
|  | rs925946 | G | T | 0.688 | 0.015 | 0.002 | 810865 | 81.779 |
|  | rs7944584 | T | A | 0.273 | 0.022 | 0.002 | 810865 | 149.031 |
|  | rs909116 | C | T | 0.479 | 0.015 | 0.002 | 810865 | 92.224 |
|  | rs11024074 | C | T | 0.301 | 0.012 | 0.002 | 810865 | 52.358 |
|  | rs11222085 | C | T | 0.189 | -0.015 | 0.002 | 810865 | 54.877 |
|  | rs11030016 | T | C | 0.733 | -0.011 | 0.002 | 810865 | 41.367 |
|  | rs2373115 | A | C | 0.165 | -0.012 | 0.002 | 810865 | 31.833 |
|  | rs1145415 | A | G | 0.469 | 0.015 | 0.002 | 810865 | 91.058 |
|  | rs415895 | G | C | 0.654 | 0.018 | 0.002 | 810865 | 123.143 |
|  | rs11229457 | T | C | 0.217 | -0.011 | 0.002 | 810865 | 33.251 |
|  | rs12801636 | A | G | 0.223 | -0.023 | 0.002 | 810865 | 143.271 |
|  | rs2186564 | A | G | 0.112 | 0.016 | 0.003 | 810865 | 41.128 |
|  | rs2926747 | T | A | 0.635 | -0.013 | 0.002 | 810865 | 60.047 |
|  | rs4883201 | G | A | 0.103 | 0.015 | 0.003 | 810865 | 34.759 |
|  | rs10743347 | A | G | 0.890 | 0.021 | 0.003 | 810865 | 72.102 |
|  | rs17287293 | G | A | 0.149 | -0.014 | 0.002 | 810865 | 38.006 |
|  | rs6581895 | C | T | 0.221 | 0.010 | 0.002 | 810865 | 30.110 |
|  | rs17249754 | A | G | 0.167 | -0.034 | 0.002 | 810865 | 265.286 |
|  | rs1060105 | T | C | 0.204 | -0.020 | 0.002 | 810865 | 103.359 |
|  | rs7134375 | A | C | 0.430 | -0.010 | 0.002 | 810865 | 42.625 |
|  | rs4587807 | A | G | 0.510 | -0.009 | 0.002 | 810865 | 32.037 |
|  | rs7302981 | G | A | 0.625 | -0.026 | 0.002 | 810865 | 262.373 |
|  | rs2292239 | G | T | 0.660 | 0.011 | 0.002 | 810865 | 42.682 |
|  | rs3184504 | C | T | 0.519 | -0.043 | 0.002 | 810865 | 751.309 |
|  | rs35444 | G | A | 0.386 | -0.023 | 0.002 | 810865 | 199.455 |
|  | rs3118905 | A | G | 0.279 | 0.011 | 0.002 | 810865 | 35.994 |
|  | rs1933437 | A | G | 0.620 | 0.009 | 0.002 | 810865 | 31.782 |
|  | rs17880989 | A | G | 0.027 | 0.032 | 0.005 | 810865 | 43.462 |
|  | rs1950500 | C | T | 0.709 | -0.013 | 0.002 | 810865 | 56.011 |
|  | rs4899260 | T | C | 0.248 | 0.012 | 0.002 | 810865 | 45.326 |
|  | rs2652834 | G | A | 0.807 | -0.011 | 0.002 | 810865 | 29.818 |
|  | rs7173826 | G | T | 0.329 | -0.010 | 0.002 | 810865 | 36.166 |
|  | rs17677991 | G | C | 0.350 | 0.016 | 0.002 | 810865 | 90.750 |
|  | rs1060939 | A | G | 0.291 | 0.016 | 0.002 | 810865 | 79.148 |
|  | rs1378942 | A | C | 0.668 | -0.034 | 0.002 | 810865 | 410.382 |
|  | rs6496047 | C | T | 0.435 | 0.013 | 0.002 | 810865 | 71.270 |
|  | rs3825807 | G | A | 0.444 | 0.011 | 0.002 | 810865 | 37.683 |
|  | rs17514846 | A | C | 0.466 | 0.026 | 0.002 | 810865 | 267.930 |
|  | rs1049205 | T | C | 0.560 | 0.015 | 0.002 | 810865 | 88.600 |
|  | rs2286472 | C | A | 0.319 | 0.013 | 0.002 | 810865 | 61.638 |
|  | rs12917707 | T | G | 0.183 | -0.025 | 0.002 | 810865 | 150.135 |
|  | rs11548855 | C | T | 0.073 | -0.018 | 0.003 | 810865 | 36.612 |
|  | rs11639856 | A | T | 0.193 | -0.011 | 0.002 | 810865 | 30.176 |
|  | rs1126464 | C | G | 0.244 | 0.020 | 0.002 | 810865 | 112.024 |
|  | rs2865531 | A | T | 0.594 | 0.011 | 0.002 | 810865 | 35.857 |
|  | rs35675346 | A | G | 0.241 | 0.015 | 0.002 | 810865 | 62.578 |
|  | rs1885987 | G | T | 0.371 | -0.011 | 0.002 | 810865 | 44.587 |
|  | rs3760318 | A | G | 0.374 | -0.009 | 0.002 | 810865 | 31.558 |
|  | rs4562 | G | A | 0.619 | 0.013 | 0.002 | 810865 | 55.811 |
|  | rs4968639 | G | A | 0.201 | -0.011 | 0.002 | 810865 | 31.360 |
|  | rs1053739 | A | G | 0.346 | 0.015 | 0.002 | 810865 | 71.209 |
|  | rs1135640 | C | G | 0.664 | 0.009 | 0.002 | 810865 | 31.513 |
|  | rs1043809 | T | C | 0.811 | 0.014 | 0.002 | 810865 | 43.901 |
|  | rs16948048 | G | A | 0.373 | 0.017 | 0.002 | 810865 | 117.198 |
|  | rs8068318 | T | C | 0.732 | 0.016 | 0.002 | 810865 | 84.586 |
|  | rs55679337 | C | T | 0.328 | 0.010 | 0.002 | 810865 | 34.662 |
|  | rs7234309 | T | C | 0.270 | -0.011 | 0.002 | 810865 | 37.905 |
|  | rs3810291 | A | G | 0.673 | -0.011 | 0.002 | 810865 | 46.523 |
|  | rs167479 | T | G | 0.471 | -0.031 | 0.002 | 810865 | 376.679 |
|  | rs1130222 | T | C | 0.409 | 0.012 | 0.002 | 810865 | 59.407 |
|  | rs7248171 | A | G | 0.401 | 0.014 | 0.002 | 810865 | 73.411 |
|  | rs2228603 | T | C | 0.077 | 0.029 | 0.003 | 810865 | 97.468 |
|  | rs8102137 | C | T | 0.330 | 0.018 | 0.002 | 810865 | 122.639 |
|  | rs814501 | A | G | 0.518 | -0.009 | 0.002 | 810865 | 33.816 |
|  | rs4803448 | C | T | 0.461 | 0.009 | 0.002 | 810865 | 33.168 |
|  | rs1887320 | A | G | 0.470 | 0.029 | 0.002 | 810865 | 354.476 |
|  | rs6077853 | A | G | 0.162 | -0.019 | 0.002 | 810865 | 80.545 |
|  | rs879597 | G | A | 0.157 | -0.015 | 0.002 | 810865 | 40.635 |
|  | rs6095241 | A | G | 0.437 | -0.014 | 0.002 | 810865 | 75.701 |
|  | rs16982520 | G | A | 0.126 | 0.043 | 0.002 | 810865 | 326.233 |
|  | rs6062343 | A | G | 0.438 | -0.011 | 0.002 | 810865 | 50.561 |
|  | rs9636639 | A | C | 0.482 | 0.011 | 0.002 | 810865 | 51.528 |
|  | rs9306160 | C | T | 0.590 | 0.013 | 0.002 | 810865 | 66.946 |
|  | rs4823006 | G | A | 0.446 | -0.014 | 0.002 | 810865 | 79.364 |
|  | rs2006771 | A | G | 0.457 | 0.009 | 0.002 | 810865 | 29.886 |
|  |  |  |  |  |  |  |  |  |
| Insomnia | rs12049261 | C | G | 0.293 | 0.011 | 0.002 | 462341 | 47.074 |
|  | rs6690017 | G | T | 0.409 | -0.010 | 0.002 | 462341 | 46.216 |
|  | rs4572538 | T | C | 0.364 | -0.010 | 0.002 | 462341 | 37.839 |
|  | rs56365214 | A | C | 0.156 | -0.015 | 0.002 | 462341 | 51.970 |
|  | rs4577309 | G | A | 0.534 | -0.009 | 0.001 | 462341 | 32.820 |
|  | rs12470989 | G | A | 0.204 | -0.010 | 0.002 | 462341 | 30.826 |
|  | rs113851554 | T | G | 0.057 | 0.047 | 0.003 | 462341 | 199.344 |
|  | rs56093896 | A | C | 0.214 | -0.012 | 0.002 | 462341 | 46.835 |
|  | rs2014830 | T | C | 0.304 | -0.012 | 0.002 | 462341 | 51.080 |
|  | rs705219 | A | T | 0.887 | 0.013 | 0.002 | 462341 | 32.543 |
|  | rs9845387 | A | C | 0.040 | -0.022 | 0.004 | 462341 | 33.494 |
|  | rs1988337 | G | A | 0.552 | 0.008 | 0.001 | 462341 | 31.413 |
|  | rs11097861 | G | A | 0.716 | 0.010 | 0.002 | 462341 | 37.077 |
|  | rs2604551 | G | T | 0.640 | -0.008 | 0.002 | 462341 | 29.846 |
|  | rs1592757 | C | G | 0.356 | 0.010 | 0.002 | 462341 | 43.491 |
|  | rs7711696 | T | G | 0.305 | 0.011 | 0.002 | 462341 | 48.065 |
|  | rs1430205 | T | C | 0.462 | 0.009 | 0.001 | 462341 | 40.379 |
|  | rs314280 | G | A | 0.547 | 0.010 | 0.001 | 462341 | 42.424 |
|  | rs6975972 | G | A | 0.579 | -0.009 | 0.002 | 462341 | 35.960 |
|  | rs17151854 | T | G | 0.152 | 0.013 | 0.002 | 462341 | 39.211 |
|  | rs11790060 | C | T | 0.331 | -0.010 | 0.002 | 462341 | 42.900 |
|  | rs224032 | A | G | 0.550 | 0.008 | 0.001 | 462341 | 31.666 |
|  | rs17709610 | G | A | 0.298 | -0.010 | 0.002 | 462341 | 37.431 |
|  | rs2297787 | A | T | 0.080 | -0.018 | 0.003 | 462341 | 41.898 |
|  | rs72924721 | T | C | 0.073 | 0.016 | 0.003 | 462341 | 32.712 |
|  | rs10838708 | A | G | 0.459 | -0.009 | 0.002 | 462341 | 39.769 |
|  | rs68094047 | T | C | 0.251 | 0.010 | 0.002 | 462341 | 36.248 |
|  | rs931221 | A | T | 0.237 | 0.011 | 0.002 | 462341 | 36.807 |
|  | rs324017 | C | A | 0.705 | -0.010 | 0.002 | 462341 | 36.693 |
|  | rs9570080 | C | T | 0.344 | -0.011 | 0.002 | 462341 | 45.413 |
|  | rs6561715 | A | T | 0.631 | -0.012 | 0.002 | 462341 | 56.790 |
|  | rs1547630 | A | G | 0.652 | 0.009 | 0.002 | 462341 | 33.894 |
|  | rs4886860 | C | G | 0.767 | -0.012 | 0.002 | 462341 | 45.143 |
|  | rs11635495 | C | T | 0.512 | 0.009 | 0.001 | 462341 | 39.837 |
|  | rs2062113 | C | T | 0.568 | -0.010 | 0.002 | 462341 | 40.953 |
|  | rs9894577 | A | G | 0.318 | 0.013 | 0.002 | 462341 | 68.382 |
|  | rs9906181 | G | A | 0.688 | -0.009 | 0.002 | 462341 | 31.180 |
|  | rs11152363 | A | G | 0.186 | 0.016 | 0.002 | 462341 | 65.992 |
|  | rs56330606 | G | A | 0.379 | 0.009 | 0.002 | 462341 | 37.000 |
|  |  |  |  |  |  |  |  |  |
| BMI | rs909001 | G | C | 0.172 | 0.016 | 0.003 | 461460 | 37.759 |
|  | rs12140153 | T | G | 0.094 | -0.033 | 0.003 | 461460 | 91.437 |
|  | rs12072739 | G | A | 0.224 | 0.016 | 0.002 | 461460 | 44.003 |
|  | rs41279738 | G | T | 0.026 | 0.068 | 0.006 | 461460 | 120.925 |
|  | rs115056380 | A | G | 0.048 | -0.026 | 0.005 | 461460 | 31.069 |
|  | rs2618039 | T | A | 0.381 | 0.014 | 0.002 | 461460 | 50.209 |
|  | rs1778830 | A | G | 0.362 | 0.014 | 0.002 | 461460 | 46.883 |
|  | rs61828641 | A | G | 0.109 | 0.022 | 0.003 | 461460 | 50.539 |
|  | rs2791643 | T | C | 0.762 | -0.013 | 0.002 | 461460 | 33.472 |
|  | rs156914 | A | G | 0.492 | 0.011 | 0.002 | 461460 | 31.964 |
|  | rs10799778 | G | T | 0.834 | -0.018 | 0.003 | 461460 | 47.335 |
|  | rs113603865 | T | C | 0.212 | 0.019 | 0.002 | 461460 | 58.747 |
|  | rs11165643 | T | C | 0.590 | 0.019 | 0.002 | 461460 | 93.116 |
|  | rs61813324 | T | C | 0.136 | 0.029 | 0.003 | 461460 | 98.785 |
|  | rs11122450 | G | T | 0.612 | -0.012 | 0.002 | 461460 | 33.018 |
|  | rs10927006 | C | T | 0.144 | -0.017 | 0.003 | 461460 | 35.692 |
|  | rs3866805 | A | C | 0.356 | 0.012 | 0.002 | 461460 | 32.547 |
|  | rs61740466 | A | G | 0.237 | -0.014 | 0.002 | 461460 | 33.971 |
|  | rs6682438 | C | T | 0.673 | 0.013 | 0.002 | 461460 | 39.245 |
|  | rs7519259 | A | G | 0.528 | 0.014 | 0.002 | 461460 | 49.823 |
|  | rs3845344 | T | C | 0.391 | 0.016 | 0.002 | 461460 | 65.648 |
|  | rs12089815 | A | G | 0.549 | -0.012 | 0.002 | 461460 | 38.431 |
|  | rs7539903 | A | T | 0.616 | -0.013 | 0.002 | 461460 | 42.196 |
|  | rs7516554 | T | C | 0.400 | 0.012 | 0.002 | 461460 | 35.482 |
|  | rs4648450 | A | C | 0.467 | -0.015 | 0.002 | 461460 | 55.714 |
|  | rs6669341 | G | A | 0.583 | -0.017 | 0.002 | 461460 | 72.595 |
|  | rs1167311 | A | G | 0.681 | -0.019 | 0.002 | 461460 | 81.720 |
|  | rs2568958 | A | G | 0.604 | 0.022 | 0.002 | 461460 | 122.759 |
|  | rs76702514 | G | C | 0.211 | -0.016 | 0.002 | 461460 | 45.923 |
|  | rs4658403 | T | C | 0.834 | -0.019 | 0.003 | 461460 | 50.801 |
|  | rs72634826 | A | G | 0.260 | -0.021 | 0.002 | 461460 | 87.085 |
|  | rs34517439 | A | C | 0.122 | 0.039 | 0.003 | 461460 | 162.250 |
|  | rs12088284 | T | C | 0.301 | 0.014 | 0.002 | 461460 | 42.045 |
|  | rs17544384 | C | T | 0.211 | 0.014 | 0.002 | 461460 | 34.073 |
|  | rs539515 | C | A | 0.205 | 0.050 | 0.002 | 461460 | 411.163 |
|  | rs815163 | C | T | 0.563 | -0.016 | 0.002 | 461460 | 68.912 |
|  | rs2678204 | G | T | 0.340 | 0.024 | 0.002 | 461460 | 134.723 |
|  | rs6744646 | G | A | 0.828 | 0.055 | 0.003 | 461460 | 450.920 |
|  | rs935166 | A | G | 0.507 | -0.016 | 0.002 | 461460 | 66.648 |
|  | rs13420048 | A | C | 0.365 | -0.015 | 0.002 | 461460 | 56.882 |
|  | rs4672338 | T | C | 0.336 | 0.014 | 0.002 | 461460 | 42.257 |
|  | rs6752979 | A | G | 0.317 | 0.013 | 0.002 | 461460 | 34.988 |
|  | rs2381404 | C | T | 0.244 | 0.014 | 0.002 | 461460 | 36.882 |
|  | rs2216931 | A | C | 0.662 | 0.017 | 0.002 | 461460 | 65.729 |
|  | rs1064213 | A | G | 0.478 | 0.015 | 0.002 | 461460 | 57.323 |
|  | rs73985439 | C | A | 0.307 | 0.014 | 0.002 | 461460 | 40.732 |
|  | rs6707827 | G | A | 0.704 | 0.012 | 0.002 | 461460 | 30.202 |
|  | rs745249 | T | C | 0.282 | 0.018 | 0.002 | 461460 | 64.247 |
|  | rs3213943 | A | C | 0.132 | -0.018 | 0.003 | 461460 | 38.761 |
|  | rs62190049 | C | G | 0.390 | -0.011 | 0.002 | 461460 | 30.147 |
|  | rs13427822 | G | A | 0.271 | -0.018 | 0.002 | 461460 | 65.581 |
|  | rs6725931 | T | C | 0.848 | 0.019 | 0.003 | 461460 | 48.473 |
|  | rs4605363 | C | A | 0.342 | 0.016 | 0.002 | 461460 | 62.052 |
|  | rs2433733 | A | G | 0.678 | -0.017 | 0.002 | 461460 | 66.357 |
|  | rs62107261 | C | T | 0.048 | -0.091 | 0.005 | 461460 | 391.190 |
|  | rs7571496 | G | A | 0.261 | -0.016 | 0.002 | 461460 | 49.684 |
|  | rs10169594 | C | T | 0.363 | 0.012 | 0.002 | 461460 | 35.170 |
|  | rs35809007 | A | G | 0.363 | -0.017 | 0.002 | 461460 | 69.163 |
|  | rs1861410 | T | C | 0.555 | -0.021 | 0.002 | 461460 | 114.184 |
|  | rs429343 | G | A | 0.577 | -0.017 | 0.002 | 461460 | 75.716 |
|  | rs62176243 | T | A | 0.245 | -0.015 | 0.002 | 461460 | 42.779 |
|  | rs6705567 | C | T | 0.376 | -0.015 | 0.002 | 461460 | 50.879 |
|  | rs6545714 | A | G | 0.601 | -0.021 | 0.002 | 461460 | 103.650 |
|  | rs2861685 | C | T | 0.412 | -0.017 | 0.002 | 461460 | 73.511 |
|  | rs4832298 | T | C | 0.686 | -0.016 | 0.002 | 461460 | 56.567 |
|  | rs11691869 | A | C | 0.362 | -0.019 | 0.002 | 461460 | 88.295 |
|  | rs13033310 | A | G | 0.253 | 0.013 | 0.002 | 461460 | 30.524 |
|  | rs56133507 | G | T | 0.197 | 0.014 | 0.002 | 461460 | 30.948 |
|  | rs11675464 | G | A | 0.563 | 0.012 | 0.002 | 461460 | 36.373 |
|  | rs10172070 | T | C | 0.146 | 0.016 | 0.003 | 461460 | 33.618 |
|  | rs6710091 | G | C | 0.348 | -0.012 | 0.002 | 461460 | 31.947 |
|  | rs56930105 | T | C | 0.139 | 0.016 | 0.003 | 461460 | 30.309 |
|  | rs13012070 | A | G | 0.228 | -0.014 | 0.002 | 461460 | 33.798 |
|  | rs6713781 | C | G | 0.402 | -0.014 | 0.002 | 461460 | 44.798 |
|  | rs10182416 | G | A | 0.512 | 0.013 | 0.002 | 461460 | 43.740 |
|  | rs6430068 | A | G | 0.109 | 0.019 | 0.003 | 461460 | 34.060 |
|  | rs12692596 | T | C | 0.372 | 0.013 | 0.002 | 461460 | 41.262 |
|  | rs34234296 | A | G | 0.392 | -0.015 | 0.002 | 461460 | 53.667 |
|  | rs4482463 | A | C | 0.923 | -0.031 | 0.004 | 461460 | 71.375 |
|  | rs7619139 | A | T | 0.589 | 0.013 | 0.002 | 461460 | 44.774 |
|  | rs11919665 | T | A | 0.680 | -0.013 | 0.002 | 461460 | 36.615 |
|  | rs9843653 | C | T | 0.512 | 0.029 | 0.002 | 461460 | 222.277 |
|  | rs9876664 | T | G | 0.375 | -0.018 | 0.002 | 461460 | 78.135 |
|  | rs1471093 | A | G | 0.617 | 0.013 | 0.002 | 461460 | 43.548 |
|  | rs1608113 | T | A | 0.365 | -0.012 | 0.002 | 461460 | 32.974 |
|  | rs529200 | G | A | 0.528 | 0.017 | 0.002 | 461460 | 72.932 |
|  | rs62241847 | G | A | 0.314 | -0.012 | 0.002 | 461460 | 34.068 |
|  | rs6777784 | T | G | 0.617 | 0.012 | 0.002 | 461460 | 32.803 |
|  | rs56038322 | A | G | 0.311 | 0.014 | 0.002 | 461460 | 42.029 |
|  | rs78605811 | C | A | 0.054 | -0.033 | 0.004 | 461460 | 54.199 |
|  | rs9835772 | T | A | 0.244 | 0.017 | 0.002 | 461460 | 52.328 |
|  | rs9830592 | A | C | 0.582 | 0.015 | 0.002 | 461460 | 59.847 |
|  | rs1471740 | C | T | 0.741 | 0.019 | 0.002 | 461460 | 73.753 |
|  | rs355777 | C | G | 0.408 | 0.015 | 0.002 | 461460 | 57.546 |
|  | rs12696039 | G | A | 0.149 | -0.015 | 0.003 | 461460 | 30.419 |
|  | rs2606228 | C | A | 0.646 | -0.014 | 0.002 | 461460 | 44.362 |
|  | rs73052033 | C | T | 0.185 | -0.030 | 0.003 | 461460 | 142.465 |
|  | rs17668356 | G | C | 0.146 | -0.023 | 0.003 | 461460 | 68.125 |
|  | rs76183894 | C | T | 0.081 | -0.022 | 0.004 | 461460 | 36.304 |
|  | rs9839081 | A | G | 0.325 | -0.012 | 0.002 | 461460 | 29.884 |
|  | rs3851998 | G | C | 0.743 | -0.014 | 0.002 | 461460 | 35.972 |
|  | rs56143236 | T | C | 0.256 | 0.013 | 0.002 | 461460 | 31.265 |
|  | rs6444950 | A | G | 0.237 | 0.016 | 0.002 | 461460 | 46.687 |
|  | rs62246311 | A | G | 0.102 | 0.021 | 0.003 | 461460 | 40.657 |
|  | rs28350 | G | A | 0.821 | -0.018 | 0.003 | 461460 | 48.768 |
|  | rs6769617 | T | A | 0.664 | -0.014 | 0.002 | 461460 | 42.230 |
|  | rs4858940 | C | T | 0.886 | 0.023 | 0.003 | 461460 | 54.711 |
|  | rs11709402 | G | A | 0.279 | 0.023 | 0.002 | 461460 | 106.811 |
|  | rs2920503 | T | C | 0.285 | -0.014 | 0.002 | 461460 | 40.797 |
|  | rs2569993 | C | T | 0.320 | 0.013 | 0.002 | 461460 | 35.649 |
|  | rs13097918 | A | T | 0.212 | 0.015 | 0.002 | 461460 | 36.296 |
|  | rs754635 | G | C | 0.887 | 0.022 | 0.003 | 461460 | 50.029 |
|  | rs4017425 | T | C | 0.470 | -0.013 | 0.002 | 461460 | 40.364 |
|  | rs2035936 | T | G | 0.056 | 0.037 | 0.004 | 461460 | 72.426 |
|  | rs6774894 | A | T | 0.358 | 0.013 | 0.002 | 461460 | 41.871 |
|  | rs2051559 | C | T | 0.133 | 0.020 | 0.003 | 461460 | 48.858 |
|  | rs1000096 | T | C | 0.401 | -0.013 | 0.002 | 461460 | 43.130 |
|  | rs72649373 | C | T | 0.143 | 0.018 | 0.003 | 461460 | 38.278 |
|  | rs4419475 | T | A | 0.407 | 0.011 | 0.002 | 461460 | 32.641 |
|  | rs13107325 | T | C | 0.075 | 0.048 | 0.004 | 461460 | 160.573 |
|  | rs1296328 | C | A | 0.559 | -0.019 | 0.002 | 461460 | 89.032 |
|  | rs66679256 | T | C | 0.446 | 0.015 | 0.002 | 461460 | 56.095 |
|  | rs73213484 | T | A | 0.141 | -0.023 | 0.003 | 461460 | 63.355 |
|  | rs59068084 | T | G | 0.410 | 0.011 | 0.002 | 461460 | 30.237 |
|  | rs11099020 | T | C | 0.641 | -0.014 | 0.002 | 461460 | 47.448 |
|  | rs111598585 | T | C | 0.209 | -0.014 | 0.002 | 461460 | 34.042 |
|  | rs7683836 | A | G | 0.557 | -0.012 | 0.002 | 461460 | 37.712 |
|  | rs6831088 | A | G | 0.640 | -0.012 | 0.002 | 461460 | 31.312 |
|  | rs34811474 | A | G | 0.231 | -0.029 | 0.002 | 461460 | 148.303 |
|  | rs923994 | G | A | 0.783 | -0.015 | 0.002 | 461460 | 36.818 |
|  | rs6843852 | T | C | 0.508 | 0.013 | 0.002 | 461460 | 43.879 |
|  | rs2102278 | G | A | 0.322 | 0.012 | 0.002 | 461460 | 31.468 |
|  | rs2192158 | G | A | 0.553 | -0.015 | 0.002 | 461460 | 57.309 |
|  | rs1346841 | A | G | 0.405 | -0.013 | 0.002 | 461460 | 41.891 |
|  | rs113079574 | T | C | 0.193 | -0.016 | 0.003 | 461460 | 38.393 |
|  | rs4261944 | G | T | 0.365 | 0.014 | 0.002 | 461460 | 45.390 |
|  | rs4148155 | G | A | 0.113 | -0.023 | 0.003 | 461460 | 54.661 |
|  | rs1229984 | C | T | 0.973 | 0.037 | 0.006 | 461460 | 38.845 |
|  | rs17289010 | G | A | 0.328 | -0.013 | 0.002 | 461460 | 40.948 |
|  | rs2307111 | C | T | 0.395 | -0.028 | 0.002 | 461460 | 191.809 |
|  | rs62379271 | G | T | 0.579 | 0.012 | 0.002 | 461460 | 34.171 |
|  | rs329118 | T | C | 0.419 | -0.017 | 0.002 | 461460 | 68.409 |
|  | rs1438945 | A | T | 0.715 | -0.013 | 0.002 | 461460 | 37.072 |
|  | rs13176429 | C | T | 0.688 | 0.014 | 0.002 | 461460 | 44.116 |
|  | rs116374395 | A | G | 0.035 | 0.032 | 0.005 | 461460 | 35.348 |
|  | rs28404639 | T | C | 0.366 | -0.012 | 0.002 | 461460 | 32.476 |
|  | rs10063055 | T | C | 0.253 | 0.014 | 0.002 | 461460 | 36.307 |
|  | rs1503526 | C | T | 0.480 | 0.015 | 0.002 | 461460 | 60.932 |
|  | rs9291822 | T | C | 0.515 | -0.014 | 0.002 | 461460 | 51.132 |
|  | rs7707394 | A | G | 0.357 | -0.019 | 0.002 | 461460 | 87.463 |
|  | rs252761 | T | G | 0.588 | -0.012 | 0.002 | 461460 | 32.511 |
|  | rs2962334 | T | G | 0.020 | 0.043 | 0.007 | 461460 | 37.800 |
|  | rs7708584 | G | A | 0.572 | -0.016 | 0.002 | 461460 | 63.783 |
|  | rs17056301 | C | T | 0.256 | 0.014 | 0.002 | 461460 | 35.815 |
|  | rs11134679 | G | A | 0.685 | 0.018 | 0.002 | 461460 | 73.182 |
|  | rs1919243 | C | T | 0.487 | 0.012 | 0.002 | 461460 | 34.022 |
|  | rs40071 | C | T | 0.180 | -0.026 | 0.003 | 461460 | 102.732 |
|  | rs1582931 | A | G | 0.473 | -0.013 | 0.002 | 461460 | 44.698 |
|  | rs2133561 | T | A | 0.611 | -0.014 | 0.002 | 461460 | 47.406 |
|  | rs698147 | G | A | 0.544 | -0.013 | 0.002 | 461460 | 41.909 |
|  | rs111689389 | C | G | 0.283 | -0.014 | 0.002 | 461460 | 38.781 |
|  | rs7442885 | G | C | 0.214 | -0.023 | 0.002 | 461460 | 89.444 |
|  | rs1477290 | C | T | 0.137 | 0.034 | 0.003 | 461460 | 135.832 |
|  | rs159037 | C | T | 0.254 | 0.012 | 0.002 | 461460 | 29.761 |
|  | rs32421 | T | A | 0.224 | 0.013 | 0.002 | 461460 | 30.971 |
|  | rs11757278 | C | T | 0.304 | -0.015 | 0.002 | 461460 | 46.355 |
|  | rs1322842 | G | A | 0.609 | -0.013 | 0.002 | 461460 | 41.608 |
|  | rs34045288 | T | C | 0.334 | 0.023 | 0.002 | 461460 | 125.748 |
|  | rs9349235 | T | C | 0.411 | 0.011 | 0.002 | 461460 | 30.966 |
|  | rs6938973 | C | T | 0.601 | 0.018 | 0.002 | 461460 | 81.480 |
|  | rs57989773 | C | T | 0.245 | 0.013 | 0.002 | 461460 | 31.908 |
|  | rs156201 | C | G | 0.753 | 0.013 | 0.002 | 461460 | 33.195 |
|  | rs2875762 | C | G | 0.243 | 0.015 | 0.002 | 461460 | 44.013 |
|  | rs6922607 | G | A | 0.190 | 0.015 | 0.003 | 461460 | 35.033 |
|  | rs75499503 | T | C | 0.220 | -0.018 | 0.002 | 461460 | 55.599 |
|  | rs62407562 | A | T | 0.269 | 0.014 | 0.002 | 461460 | 42.128 |
|  | rs2814942 | A | G | 0.330 | 0.028 | 0.002 | 461460 | 184.404 |
|  | rs1327259 | G | A | 0.388 | -0.015 | 0.002 | 461460 | 53.345 |
|  | rs1266874 | G | A | 0.350 | 0.014 | 0.002 | 461460 | 46.322 |
|  | rs7776021 | A | G | 0.288 | 0.012 | 0.002 | 461460 | 32.124 |
|  | rs6909685 | T | C | 0.327 | -0.015 | 0.002 | 461460 | 47.797 |
|  | rs7762794 | G | A | 0.285 | 0.015 | 0.002 | 461460 | 46.505 |
|  | rs72892910 | T | G | 0.172 | 0.039 | 0.003 | 461460 | 218.993 |
|  | rs13218383 | G | C | 0.335 | -0.014 | 0.002 | 461460 | 47.381 |
|  | rs2248551 | A | G | 0.165 | 0.015 | 0.003 | 461460 | 30.229 |
|  | rs28366156 | C | T | 0.131 | -0.026 | 0.003 | 461460 | 81.705 |
|  | rs9267671 | A | G | 0.061 | 0.026 | 0.004 | 461460 | 39.974 |
|  | rs7761673 | A | T | 0.220 | -0.014 | 0.002 | 461460 | 32.337 |
|  | rs9478496 | C | T | 0.164 | 0.018 | 0.003 | 461460 | 46.210 |
|  | rs9463175 | T | C | 0.339 | -0.012 | 0.002 | 461460 | 29.956 |
|  | rs9294260 | A | G | 0.477 | 0.015 | 0.002 | 461460 | 55.278 |
|  | rs2253310 | G | C | 0.626 | 0.017 | 0.002 | 461460 | 72.013 |
|  | rs36007635 | A | G | 0.138 | -0.021 | 0.003 | 461460 | 53.849 |
|  | rs4722398 | T | C | 0.136 | 0.019 | 0.003 | 461460 | 42.290 |
|  | rs11525873 | C | T | 0.098 | -0.024 | 0.003 | 461460 | 51.662 |
|  | rs1805123 | G | T | 0.245 | -0.017 | 0.002 | 461460 | 53.214 |
|  | rs58862095 | T | C | 0.419 | -0.023 | 0.002 | 461460 | 131.076 |
|  | rs34696181 | C | T | 0.476 | 0.011 | 0.002 | 461460 | 33.255 |
|  | rs6962980 | C | A | 0.556 | -0.016 | 0.002 | 461460 | 64.416 |
|  | rs6950388 | A | G | 0.795 | 0.016 | 0.002 | 461460 | 40.202 |
|  | rs3807566 | T | G | 0.438 | -0.012 | 0.002 | 461460 | 36.590 |
|  | rs74750282 | C | T | 0.087 | -0.020 | 0.004 | 461460 | 31.027 |
|  | rs17132130 | C | G | 0.221 | -0.018 | 0.002 | 461460 | 55.933 |
|  | rs9638713 | G | A | 0.975 | -0.036 | 0.006 | 461460 | 32.411 |
|  | rs213518 | C | T | 0.146 | 0.016 | 0.003 | 461460 | 31.688 |
|  | rs2289379 | T | C | 0.396 | -0.015 | 0.002 | 461460 | 56.658 |
|  | rs73124396 | C | T | 0.205 | -0.015 | 0.002 | 461460 | 39.597 |
|  | rs3901286 | A | C | 0.152 | -0.023 | 0.003 | 461460 | 66.978 |
|  | rs4307239 | G | A | 0.459 | 0.012 | 0.002 | 461460 | 37.293 |
|  | rs215634 | G | A | 0.612 | -0.016 | 0.002 | 461460 | 58.186 |
|  | rs17149254 | C | T | 0.805 | -0.021 | 0.003 | 461460 | 69.664 |
|  | rs7805441 | T | C | 0.502 | 0.013 | 0.002 | 461460 | 45.224 |
|  | rs7802342 | G | T | 0.289 | 0.012 | 0.002 | 461460 | 31.662 |
|  | rs13248187 | C | T | 0.269 | 0.016 | 0.002 | 461460 | 49.458 |
|  | rs36061954 | T | C | 0.399 | 0.013 | 0.002 | 461460 | 40.461 |
|  | rs12541408 | C | T | 0.317 | -0.014 | 0.002 | 461460 | 45.355 |
|  | rs12681792 | A | C | 0.193 | 0.015 | 0.003 | 461460 | 34.901 |
|  | rs1078141 | T | C | 0.384 | 0.014 | 0.002 | 461460 | 47.740 |
|  | rs2725371 | G | A | 0.696 | -0.016 | 0.002 | 461460 | 55.155 |
|  | rs11778219 | G | A | 0.163 | 0.016 | 0.003 | 461460 | 34.548 |
|  | rs4876611 | G | A | 0.720 | 0.020 | 0.002 | 461460 | 80.264 |
|  | rs72673947 | G | A | 0.107 | 0.022 | 0.003 | 461460 | 46.344 |
|  | rs2922757 | T | A | 0.597 | 0.012 | 0.002 | 461460 | 36.782 |
|  | rs2616143 | A | G | 0.320 | -0.014 | 0.002 | 461460 | 42.607 |
|  | rs56893062 | G | T | 0.303 | 0.013 | 0.002 | 461460 | 33.758 |
|  | rs35957544 | T | G | 0.574 | -0.020 | 0.002 | 461460 | 96.039 |
|  | rs145981104 | G | A | 0.064 | 0.023 | 0.004 | 461460 | 31.515 |
|  | rs1609010 | G | A | 0.566 | 0.021 | 0.002 | 461460 | 110.419 |
|  | rs10099330 | G | A | 0.453 | 0.012 | 0.002 | 461460 | 39.018 |
|  | rs10965698 | T | C | 0.370 | -0.011 | 0.002 | 461460 | 30.186 |
|  | rs17770336 | T | C | 0.322 | 0.024 | 0.002 | 461460 | 132.354 |
|  | rs10809621 | G | C | 0.350 | -0.013 | 0.002 | 461460 | 36.628 |
|  | rs10756714 | G | A | 0.444 | -0.021 | 0.002 | 461460 | 108.658 |
|  | rs10756792 | T | C | 0.743 | -0.019 | 0.002 | 461460 | 70.363 |
|  | rs7024334 | G | T | 0.779 | -0.014 | 0.002 | 461460 | 33.611 |
|  | rs7027304 | T | C | 0.653 | 0.015 | 0.002 | 461460 | 48.598 |
|  | rs28670671 | C | T | 0.286 | -0.012 | 0.002 | 461460 | 30.300 |
|  | rs17218879 | G | C | 0.339 | 0.013 | 0.002 | 461460 | 36.369 |
|  | rs16916303 | G | A | 0.120 | -0.019 | 0.003 | 461460 | 39.027 |
|  | rs10780248 | A | G | 0.559 | -0.012 | 0.002 | 461460 | 36.959 |
|  | rs2398861 | G | A | 0.259 | 0.018 | 0.002 | 461460 | 62.978 |
|  | rs7038943 | C | T | 0.339 | -0.014 | 0.002 | 461460 | 45.155 |
|  | rs56203622 | C | T | 0.146 | 0.018 | 0.003 | 461460 | 41.167 |
|  | rs12001437 | C | T | 0.368 | 0.012 | 0.002 | 461460 | 35.298 |
|  | rs7034554 | G | A | 0.374 | -0.013 | 0.002 | 461460 | 40.401 |
|  | rs1360201 | T | C | 0.482 | 0.013 | 0.002 | 461460 | 43.268 |
|  | rs13291723 | A | G | 0.571 | 0.011 | 0.002 | 461460 | 30.463 |
|  | rs2482356 | C | T | 0.429 | -0.011 | 0.002 | 461460 | 32.371 |
|  | rs10989067 | A | G | 0.316 | 0.017 | 0.002 | 461460 | 63.725 |
|  | rs10760277 | T | C | 0.385 | 0.014 | 0.002 | 461460 | 46.329 |
|  | rs1330199 | T | G | 0.483 | -0.012 | 0.002 | 461460 | 34.994 |
|  | rs7357754 | G | A | 0.500 | 0.014 | 0.002 | 461460 | 50.631 |
|  | rs7893571 | T | G | 0.666 | 0.014 | 0.002 | 461460 | 44.731 |
|  | rs71495038 | A | G | 0.077 | 0.028 | 0.004 | 461460 | 56.157 |
|  | rs10824211 | T | C | 0.139 | 0.021 | 0.003 | 461460 | 52.502 |
|  | rs11001963 | T | C | 0.581 | 0.012 | 0.002 | 461460 | 33.456 |
|  | rs2439823 | G | A | 0.546 | 0.019 | 0.002 | 461460 | 92.985 |
|  | rs79780963 | T | C | 0.077 | 0.024 | 0.004 | 461460 | 41.052 |
|  | rs67609008 | C | T | 0.284 | 0.017 | 0.002 | 461460 | 60.203 |
|  | rs117118217 | C | G | 0.018 | 0.045 | 0.008 | 461460 | 32.349 |
|  | rs11009685 | T | C | 0.244 | -0.013 | 0.002 | 461460 | 32.144 |
|  | rs7070670 | T | C | 0.328 | -0.012 | 0.002 | 461460 | 33.835 |
|  | rs17399739 | G | A | 0.069 | 0.027 | 0.004 | 461460 | 47.935 |
|  | rs10510025 | T | C | 0.247 | 0.018 | 0.002 | 461460 | 58.376 |
|  | rs7081254 | C | T | 0.206 | -0.014 | 0.002 | 461460 | 33.778 |
|  | rs11012732 | G | A | 0.332 | 0.022 | 0.002 | 461460 | 106.085 |
|  | rs147568678 | C | T | 0.238 | -0.013 | 0.002 | 461460 | 32.734 |
|  | rs73601548 | T | C | 0.115 | 0.018 | 0.003 | 461460 | 32.387 |
|  | rs705158 | A | T | 0.245 | 0.016 | 0.002 | 461460 | 45.797 |
|  | rs2172131 | C | T | 0.579 | -0.015 | 0.002 | 461460 | 55.572 |
|  | rs12259464 | A | G | 0.484 | 0.013 | 0.002 | 461460 | 43.402 |
|  | rs7924036 | T | G | 0.503 | -0.014 | 0.002 | 461460 | 52.157 |
|  | rs61871615 | T | C | 0.092 | -0.027 | 0.004 | 461460 | 55.297 |
|  | rs146569428 | A | G | 0.201 | 0.014 | 0.002 | 461460 | 31.510 |
|  | rs6265 | T | C | 0.188 | -0.040 | 0.003 | 461460 | 249.500 |
|  | rs558887 | G | A | 0.308 | -0.013 | 0.002 | 461460 | 36.587 |
|  | rs10742752 | C | T | 0.612 | 0.012 | 0.002 | 461460 | 33.790 |
|  | rs55707359 | G | T | 0.015 | 0.053 | 0.008 | 461460 | 42.771 |
|  | rs2234458 | T | C | 0.640 | -0.020 | 0.002 | 461460 | 98.316 |
|  | rs7928320 | G | C | 0.057 | 0.024 | 0.004 | 461460 | 32.261 |
|  | rs7925100 | A | G | 0.396 | 0.015 | 0.002 | 461460 | 53.028 |
|  | rs11218510 | A | G | 0.400 | -0.014 | 0.002 | 461460 | 51.193 |
|  | rs7944782 | G | T | 0.510 | 0.016 | 0.002 | 461460 | 62.884 |
|  | rs12364470 | G | T | 0.165 | 0.019 | 0.003 | 461460 | 52.255 |
|  | rs4929923 | C | T | 0.645 | 0.019 | 0.002 | 461460 | 84.197 |
|  | rs55769038 | A | G | 0.590 | 0.016 | 0.002 | 461460 | 64.067 |
|  | rs13642 | T | A | 0.361 | -0.016 | 0.002 | 461460 | 61.540 |
|  | rs2512892 | C | T | 0.566 | 0.013 | 0.002 | 461460 | 41.981 |
|  | rs1793636 | C | G | 0.309 | -0.013 | 0.002 | 461460 | 38.796 |
|  | rs7124681 | A | C | 0.408 | 0.026 | 0.002 | 461460 | 164.057 |
|  | rs594024 | C | T | 0.554 | -0.015 | 0.002 | 461460 | 54.352 |
|  | rs10160769 | C | G | 0.218 | -0.016 | 0.002 | 461460 | 41.413 |
|  | rs11607476 | C | A | 0.487 | 0.016 | 0.002 | 461460 | 62.226 |
|  | rs12273545 | T | C | 0.057 | 0.025 | 0.004 | 461460 | 33.595 |
|  | rs329651 | T | G | 0.804 | 0.016 | 0.003 | 461460 | 39.484 |
|  | rs59227842 | G | A | 0.311 | 0.023 | 0.002 | 461460 | 113.785 |
|  | rs7947143 | A | G | 0.163 | -0.018 | 0.003 | 461460 | 46.682 |
|  | rs10832778 | G | C | 0.623 | 0.012 | 0.002 | 461460 | 32.131 |
|  | rs349071 | A | G | 0.500 | -0.013 | 0.002 | 461460 | 45.057 |
|  | rs61903695 | G | A | 0.255 | 0.017 | 0.002 | 461460 | 53.574 |
|  | rs723672 | T | C | 0.432 | 0.011 | 0.002 | 461460 | 30.739 |
|  | rs10505836 | C | A | 0.860 | 0.018 | 0.003 | 461460 | 41.459 |
|  | rs78086698 | C | T | 0.040 | 0.032 | 0.005 | 461460 | 39.334 |
|  | rs1458156 | T | C | 0.488 | 0.014 | 0.002 | 461460 | 50.557 |
|  | rs55726687 | A | G | 0.210 | 0.025 | 0.002 | 461460 | 104.730 |
|  | rs1126930 | C | G | 0.035 | 0.032 | 0.005 | 461460 | 36.260 |
|  | rs317656 | A | T | 0.724 | -0.014 | 0.002 | 461460 | 42.790 |
|  | rs4267103 | C | T | 0.186 | 0.015 | 0.003 | 461460 | 36.630 |
|  | rs11115160 | A | G | 0.238 | -0.013 | 0.002 | 461460 | 31.326 |
|  | rs704061 | C | T | 0.455 | 0.015 | 0.002 | 461460 | 54.347 |
|  | rs55966114 | T | C | 0.193 | 0.015 | 0.003 | 461460 | 33.689 |
|  | rs147730268 | T | G | 0.087 | -0.035 | 0.004 | 461460 | 95.826 |
|  | rs6560906 | C | T | 0.692 | -0.012 | 0.002 | 461460 | 32.449 |
|  | rs7132908 | A | G | 0.384 | 0.030 | 0.002 | 461460 | 214.589 |
|  | rs2271189 | A | G | 0.403 | -0.016 | 0.002 | 461460 | 65.267 |
|  | rs4764949 | G | A | 0.326 | -0.018 | 0.002 | 461460 | 75.840 |
|  | rs73193736 | G | A | 0.244 | -0.018 | 0.002 | 461460 | 58.314 |
|  | rs3897102 | T | C | 0.411 | 0.012 | 0.002 | 461460 | 35.533 |
|  | rs7306534 | A | G | 0.622 | -0.011 | 0.002 | 461460 | 30.123 |
|  | rs11610621 | A | T | 0.148 | 0.016 | 0.003 | 461460 | 35.091 |
|  | rs4477562 | T | C | 0.129 | 0.030 | 0.003 | 461460 | 98.733 |
|  | rs4055791 | T | C | 0.417 | -0.018 | 0.002 | 461460 | 78.651 |
|  | rs56399737 | T | C | 0.449 | -0.016 | 0.002 | 461460 | 65.295 |
|  | rs17446299 | G | C | 0.166 | 0.015 | 0.003 | 461460 | 33.058 |
|  | rs1441264 | A | G | 0.594 | 0.018 | 0.002 | 461460 | 75.639 |
|  | rs9515446 | G | A | 0.448 | 0.015 | 0.002 | 461460 | 57.585 |
|  | rs1967772 | A | G | 0.285 | -0.017 | 0.002 | 461460 | 59.811 |
|  | rs9571687 | A | C | 0.329 | -0.014 | 0.002 | 461460 | 41.195 |
|  | rs56858768 | A | G | 0.297 | 0.016 | 0.002 | 461460 | 53.628 |
|  | rs7996639 | A | G | 0.449 | 0.015 | 0.002 | 461460 | 52.839 |
|  | rs7331420 | A | G | 0.285 | -0.014 | 0.002 | 461460 | 42.634 |
|  | rs9888533 | T | C | 0.538 | 0.012 | 0.002 | 461460 | 35.376 |
|  | rs9522180 | T | C | 0.553 | -0.014 | 0.002 | 461460 | 50.107 |
|  | rs11842871 | T | G | 0.260 | -0.015 | 0.002 | 461460 | 44.298 |
|  | rs6561937 | A | T | 0.754 | -0.016 | 0.002 | 461460 | 47.850 |
|  | rs217672 | C | A | 0.272 | 0.017 | 0.002 | 461460 | 58.197 |
|  | rs113624107 | A | G | 0.226 | 0.015 | 0.002 | 461460 | 40.346 |
|  | rs1286058 | A | T | 0.704 | 0.015 | 0.002 | 461460 | 47.260 |
|  | rs12881629 | G | A | 0.083 | 0.022 | 0.004 | 461460 | 37.825 |
|  | rs8015400 | A | C | 0.677 | 0.021 | 0.002 | 461460 | 101.624 |
|  | rs2383377 | A | G | 0.131 | 0.016 | 0.003 | 461460 | 30.185 |
|  | rs35697587 | A | G | 0.508 | -0.016 | 0.002 | 461460 | 69.126 |
|  | rs8020365 | A | T | 0.220 | 0.025 | 0.002 | 461460 | 109.877 |
|  | rs61992671 | G | A | 0.492 | -0.016 | 0.002 | 461460 | 61.236 |
|  | rs3803286 | G | A | 0.667 | -0.019 | 0.002 | 461460 | 78.930 |
|  | rs12889639 | A | G | 0.651 | 0.016 | 0.002 | 461460 | 55.893 |
|  | rs1451963 | T | G | 0.082 | 0.022 | 0.004 | 461460 | 37.908 |
|  | rs6575340 | A | G | 0.636 | 0.021 | 0.002 | 461460 | 101.112 |
|  | rs3902951 | G | T | 0.237 | 0.014 | 0.002 | 461460 | 35.928 |
|  | rs10144067 | T | C | 0.591 | 0.019 | 0.002 | 461460 | 83.766 |
|  | rs34153025 | C | T | 0.022 | -0.039 | 0.007 | 461460 | 32.929 |
|  | rs3784710 | C | T | 0.227 | -0.030 | 0.002 | 461460 | 158.390 |
|  | rs2870111 | T | C | 0.412 | -0.016 | 0.002 | 461460 | 60.577 |
|  | rs11630647 | A | G | 0.252 | -0.013 | 0.002 | 461460 | 30.878 |
|  | rs1657930 | A | G | 0.803 | -0.015 | 0.002 | 461460 | 34.888 |
|  | rs140159717 | T | C | 0.082 | -0.025 | 0.004 | 461460 | 44.290 |
|  | rs62007782 | A | G | 0.265 | -0.017 | 0.002 | 461460 | 55.480 |
|  | rs62020775 | A | T | 0.142 | -0.017 | 0.003 | 461460 | 33.471 |
|  | rs8025516 | G | T | 0.646 | -0.015 | 0.002 | 461460 | 49.849 |
|  | rs8024137 | T | A | 0.848 | 0.016 | 0.003 | 461460 | 31.902 |
|  | rs35697691 | G | C | 0.089 | 0.023 | 0.004 | 461460 | 42.753 |
|  | rs28568418 | A | G | 0.108 | -0.018 | 0.003 | 461460 | 32.476 |
|  | rs4284600 | C | T | 0.467 | 0.012 | 0.002 | 461460 | 35.981 |
|  | rs35364449 | T | C | 0.110 | 0.022 | 0.003 | 461460 | 46.497 |
|  | rs4444317 | G | A | 0.216 | -0.016 | 0.002 | 461460 | 44.555 |
|  | rs12440603 | T | C | 0.434 | 0.014 | 0.002 | 461460 | 48.447 |
|  | rs2899644 | T | C | 0.230 | 0.015 | 0.002 | 461460 | 40.184 |
|  | rs879620 | T | C | 0.613 | 0.024 | 0.002 | 461460 | 140.277 |
|  | rs56094641 | G | A | 0.405 | 0.073 | 0.002 | 461460 | 1331.567 |
|  | rs12149660 | A | G | 0.115 | -0.023 | 0.003 | 461460 | 53.234 |
|  | rs7206608 | G | C | 0.322 | 0.014 | 0.002 | 461460 | 40.702 |
|  | rs2075466 | C | G | 0.267 | 0.013 | 0.002 | 461460 | 35.320 |
|  | rs3814883 | T | C | 0.482 | 0.024 | 0.002 | 461460 | 146.426 |
|  | rs117342986 | T | C | 0.026 | 0.037 | 0.006 | 461460 | 31.986 |
|  | rs862320 | T | C | 0.410 | -0.023 | 0.002 | 461460 | 132.428 |
|  | rs9673839 | G | A | 0.491 | 0.013 | 0.002 | 461460 | 42.991 |
|  | rs11641179 | G | A | 0.257 | -0.013 | 0.002 | 461460 | 30.449 |
|  | rs7498665 | G | A | 0.400 | 0.027 | 0.002 | 461460 | 176.876 |
|  | rs12921986 | G | A | 0.078 | 0.020 | 0.004 | 461460 | 30.226 |
|  | rs5011579 | G | C | 0.715 | 0.014 | 0.002 | 461460 | 40.903 |
|  | rs11642090 | C | T | 0.374 | 0.011 | 0.002 | 461460 | 30.746 |
|  | rs7201895 | A | G | 0.354 | -0.015 | 0.002 | 461460 | 51.855 |
|  | rs9926784 | C | T | 0.185 | -0.024 | 0.003 | 461460 | 87.464 |
|  | rs2342892 | G | T | 0.516 | -0.013 | 0.002 | 461460 | 41.242 |
|  | rs35154326 | G | A | 0.274 | -0.013 | 0.002 | 461460 | 34.072 |
|  | rs7774 | A | C | 0.310 | 0.015 | 0.002 | 461460 | 48.521 |
|  | rs8076669 | C | T | 0.562 | 0.014 | 0.002 | 461460 | 49.676 |
|  | rs11656076 | A | G | 0.225 | -0.015 | 0.002 | 461460 | 42.360 |
|  | rs11150745 | G | A | 0.318 | -0.021 | 0.002 | 461460 | 98.736 |
|  | rs4790292 | A | C | 0.154 | -0.025 | 0.003 | 461460 | 85.278 |
|  | rs118136827 | T | G | 0.281 | -0.013 | 0.002 | 461460 | 36.255 |
|  | rs80135274 | T | A | 0.070 | 0.021 | 0.004 | 461460 | 30.229 |
|  | rs56161855 | T | A | 0.133 | 0.022 | 0.003 | 461460 | 59.238 |
|  | rs62072006 | C | A | 0.145 | 0.016 | 0.003 | 461460 | 30.714 |
|  | rs3935190 | A | G | 0.537 | -0.014 | 0.002 | 461460 | 52.621 |
|  | rs12937411 | T | C | 0.408 | -0.017 | 0.002 | 461460 | 72.837 |
|  | rs9674487 | G | C | 0.001 | 0.158 | 0.029 | 461460 | 30.655 |
|  | rs11079849 | T | C | 0.329 | -0.020 | 0.002 | 461460 | 90.542 |
|  | rs1320251 | T | C | 0.455 | -0.018 | 0.002 | 461460 | 81.773 |
|  | rs7218014 | C | T | 0.197 | 0.019 | 0.002 | 461460 | 57.833 |
|  | rs1788808 | G | A | 0.495 | -0.020 | 0.002 | 461460 | 105.921 |
|  | rs9951619 | G | T | 0.767 | 0.014 | 0.002 | 461460 | 37.421 |
|  | rs8089514 | A | T | 0.369 | 0.013 | 0.002 | 461460 | 39.145 |
|  | rs512121 | C | T | 0.192 | -0.016 | 0.003 | 461460 | 39.934 |
|  | rs784257 | C | T | 0.813 | 0.018 | 0.003 | 461460 | 49.461 |
|  | rs57636386 | C | T | 0.084 | -0.041 | 0.004 | 461460 | 132.556 |
|  | rs7232171 | T | G | 0.583 | 0.012 | 0.002 | 461460 | 37.761 |
|  | rs1834144 | A | C | 0.373 | -0.014 | 0.002 | 461460 | 46.616 |
|  | rs6567160 | C | T | 0.233 | 0.054 | 0.002 | 461460 | 534.972 |
|  | rs60764613 | T | G | 0.145 | 0.021 | 0.003 | 461460 | 55.133 |
|  | rs559231 | T | G | 0.393 | 0.013 | 0.002 | 461460 | 43.921 |
|  | rs55714539 | C | A | 0.344 | 0.018 | 0.002 | 461460 | 69.987 |
|  | rs12459368 | G | A | 0.268 | -0.017 | 0.002 | 461460 | 58.089 |
|  | rs3764625 | G | T | 0.588 | -0.012 | 0.002 | 461460 | 34.120 |
|  | rs45486197 | A | G | 0.066 | 0.026 | 0.004 | 461460 | 40.728 |
|  | rs72976986 | A | G | 0.190 | -0.023 | 0.003 | 461460 | 83.145 |
|  | rs56352336 | C | T | 0.155 | -0.016 | 0.003 | 461460 | 35.244 |
|  | rs7250833 | T | C | 0.289 | 0.014 | 0.002 | 461460 | 38.092 |
|  | rs10423928 | A | T | 0.194 | -0.034 | 0.002 | 461460 | 185.301 |
|  | rs7259070 | C | T | 0.596 | 0.022 | 0.002 | 461460 | 115.340 |
|  | rs12462975 | A | G | 0.330 | 0.020 | 0.002 | 461460 | 85.263 |
|  | rs73026725 | A | C | 0.154 | -0.022 | 0.003 | 461460 | 65.838 |
|  | rs12974458 | T | C | 0.543 | 0.015 | 0.002 | 461460 | 58.279 |
|  | rs8112818 | G | A | 0.400 | -0.021 | 0.002 | 461460 | 104.235 |
|  | rs429358 | C | T | 0.154 | -0.027 | 0.003 | 461460 | 94.501 |
|  | rs852042 | G | A | 0.759 | -0.013 | 0.002 | 461460 | 32.176 |
|  | rs73142879 | T | C | 0.192 | -0.027 | 0.003 | 461460 | 111.976 |
|  | rs2153740 | G | A | 0.480 | -0.011 | 0.002 | 461460 | 31.888 |
|  | rs2425816 | A | G | 0.415 | 0.012 | 0.002 | 461460 | 36.973 |
|  | rs6023655 | G | A | 0.766 | -0.015 | 0.002 | 461460 | 39.314 |
|  | rs11699828 | A | G | 0.036 | -0.034 | 0.006 | 461460 | 33.137 |
|  | rs34481751 | A | C | 0.165 | -0.019 | 0.003 | 461460 | 46.822 |
|  | rs1884897 | G | A | 0.627 | 0.020 | 0.002 | 461460 | 94.604 |
|  | rs4456769 | T | C | 0.333 | 0.015 | 0.002 | 461460 | 48.345 |
|  | rs909892 | A | G | 0.135 | -0.018 | 0.003 | 461460 | 39.956 |
|  | rs2837996 | C | T | 0.651 | 0.013 | 0.002 | 461460 | 37.124 |
|  | rs8132491 | A | G | 0.313 | -0.015 | 0.002 | 461460 | 49.146 |
|  | rs394608 | C | T | 0.538 | 0.019 | 0.002 | 461460 | 87.268 |
|  | rs406388 | G | C | 0.177 | 0.016 | 0.003 | 461460 | 37.689 |
|  | rs4820410 | G | A | 0.345 | -0.018 | 0.002 | 461460 | 72.420 |
|  | rs28489620 | A | G | 0.290 | -0.015 | 0.002 | 461460 | 48.821 |
|  |  |  |  |  |  |  |  |  |
| TC | rs11802413 | T | C | 0.537 | 0.029 | 0.004 | 187138 | 67.239 |
|  | rs11591147 | T | G | 0.017 | -0.334 | 0.017 | 85729 | 372.950 |
|  | rs646776 | T | C | 0.788 | 0.127 | 0.004 | 187288 | 917.215 |
|  | rs558971 | G | A | 0.530 | 0.040 | 0.004 | 187254 | 122.224 |
|  | rs2642438 | G | A | 0.745 | 0.037 | 0.004 | 179599 | 85.562 |
|  | rs7534572 | G | C | 0.690 | 0.063 | 0.006 | 83151 | 130.787 |
|  | rs6603981 | T | C | 0.806 | 0.035 | 0.004 | 187329 | 66.630 |
|  | rs7551981 | T | G | 0.595 | 0.036 | 0.004 | 187282 | 93.618 |
|  | rs4988235 | A | G | 0.524 | -0.031 | 0.004 | 183761 | 59.289 |
|  | rs11694172 | G | A | 0.216 | 0.028 | 0.004 | 187092 | 45.644 |
|  | rs2030746 | T | C | 0.398 | 0.020 | 0.004 | 187289 | 28.927 |
|  | rs17526895 | G | A | 0.078 | -0.042 | 0.007 | 184199 | 39.296 |
|  | rs2287623 | A | G | 0.595 | -0.027 | 0.004 | 184257 | 57.506 |
|  | rs9306897 | C | T | 0.697 | -0.049 | 0.004 | 185808 | 173.953 |
|  | rs780093 | C | T | 0.587 | -0.052 | 0.004 | 186446 | 204.647 |
|  | rs6544713 | C | T | 0.706 | -0.077 | 0.004 | 187199 | 373.452 |
|  | rs6709904 | G | A | 0.114 | -0.055 | 0.008 | 94558 | 43.115 |
|  | rs11563251 | T | C | 0.125 | 0.037 | 0.006 | 187107 | 38.903 |
|  | rs515135 | C | T | 0.782 | 0.124 | 0.005 | 187291 | 724.304 |
|  | rs13315871 | A | G | 0.080 | -0.036 | 0.006 | 187287 | 33.868 |
|  | rs7616006 | G | A | 0.445 | -0.032 | 0.004 | 187246 | 76.562 |
|  | rs7640978 | T | C | 0.106 | -0.038 | 0.007 | 186485 | 32.455 |
|  | rs6818397 | G | T | 0.587 | -0.025 | 0.004 | 186903 | 42.416 |
|  | rs4530754 | A | G | 0.582 | 0.023 | 0.004 | 187272 | 42.435 |
|  | rs12916 | C | T | 0.431 | 0.068 | 0.004 | 182530 | 360.996 |
|  | rs6882076 | C | T | 0.666 | 0.051 | 0.004 | 187270 | 188.503 |
|  | rs2814982 | T | C | 0.107 | -0.044 | 0.006 | 187263 | 59.858 |
|  | rs3757354 | T | C | 0.210 | -0.035 | 0.004 | 187247 | 68.652 |
|  | rs9272775 | C | T | 0.282 | 0.032 | 0.006 | 89534 | 33.219 |
|  | rs11153594 | T | C | 0.392 | -0.029 | 0.004 | 187230 | 64.891 |
|  | rs2315065 | A | C | 0.087 | 0.110 | 0.016 | 68430 | 48.645 |
|  | rs1800562 | A | G | 0.046 | -0.057 | 0.008 | 185469 | 53.841 |
|  | rs9391858 | G | A | 0.194 | 0.050 | 0.005 | 176743 | 98.009 |
|  | rs9376090 | C | T | 0.272 | -0.025 | 0.004 | 187263 | 40.322 |
|  | rs112201728 | T | C | 0.058 | 0.058 | 0.010 | 92668 | 34.441 |
|  | rs11753995 | A | G | 0.146 | 0.049 | 0.005 | 187264 | 103.784 |
|  | rs1997243 | G | A | 0.131 | 0.033 | 0.005 | 183314 | 44.089 |
|  | rs12670798 | C | T | 0.224 | 0.036 | 0.004 | 187287 | 78.819 |
|  | rs2073547 | G | A | 0.194 | 0.046 | 0.005 | 184098 | 94.130 |
|  | rs2737252 | A | G | 0.256 | -0.033 | 0.004 | 187202 | 72.031 |
|  | rs9987289 | G | A | 0.925 | 0.084 | 0.006 | 173502 | 178.623 |
|  | rs7832643 | T | G | 0.405 | 0.029 | 0.004 | 178980 | 61.008 |
|  | rs10088180 | G | A | 0.678 | -0.023 | 0.004 | 187142 | 32.490 |
|  | rs4738684 | G | A | 0.648 | -0.039 | 0.004 | 187285 | 112.244 |
|  | rs1883025 | T | C | 0.243 | -0.067 | 0.004 | 186557 | 255.236 |
|  | rs581080 | C | G | 0.821 | 0.038 | 0.005 | 187121 | 64.340 |
|  | rs11789603 | T | C | 0.090 | 0.043 | 0.006 | 186565 | 47.432 |
|  | rs2066714 | C | T | 0.120 | 0.044 | 0.008 | 93811 | 33.823 |
|  | rs579459 | C | T | 0.215 | 0.062 | 0.004 | 186925 | 198.552 |
|  | rs3780181 | G | A | 0.053 | -0.044 | 0.007 | 186134 | 38.755 |
|  | rs12412743 | T | C | 0.153 | -0.030 | 0.005 | 187282 | 40.201 |
|  | rs10904908 | G | A | 0.454 | 0.025 | 0.004 | 187112 | 48.225 |
|  | rs2255141 | G | A | 0.681 | -0.031 | 0.004 | 187266 | 64.822 |
|  | rs10900221 | A | G | 0.273 | 0.026 | 0.004 | 186785 | 38.682 |
|  | rs1535 | G | A | 0.363 | -0.050 | 0.004 | 182527 | 180.428 |
|  | rs4752805 | G | A | 0.247 | 0.025 | 0.004 | 187233 | 37.478 |
|  | rs10832962 | T | C | 0.719 | 0.032 | 0.004 | 187161 | 65.236 |
|  | rs11220462 | A | G | 0.143 | 0.047 | 0.006 | 156953 | 66.787 |
|  | rs3184504 | C | T | 0.534 | 0.032 | 0.004 | 177714 | 73.866 |
|  | rs10773003 | A | G | 0.088 | 0.037 | 0.006 | 187101 | 40.475 |
|  | rs4883201 | G | A | 0.114 | -0.035 | 0.006 | 187211 | 39.062 |
|  | rs2244608 | G | A | 0.339 | 0.031 | 0.004 | 187223 | 71.562 |
|  | rs6573778 | C | T | 0.529 | -0.026 | 0.004 | 187079 | 45.476 |
|  | rs10468017 | T | C | 0.276 | 0.062 | 0.004 | 181378 | 237.928 |
|  | rs633695 | G | A | 0.285 | 0.043 | 0.006 | 93067 | 55.733 |
|  | rs2000999 | A | G | 0.185 | 0.062 | 0.004 | 185692 | 196.635 |
|  | rs247616 | T | C | 0.293 | 0.050 | 0.004 | 185621 | 155.624 |
|  | rs6504872 | T | C | 0.472 | 0.025 | 0.004 | 185712 | 51.020 |
|  | rs2886232 | C | T | 0.880 | -0.036 | 0.006 | 176571 | 33.341 |
|  | rs314253 | C | T | 0.335 | -0.023 | 0.004 | 183868 | 39.656 |
|  | rs2156552 | T | A | 0.822 | 0.057 | 0.005 | 183439 | 147.079 |
|  | rs2738459 | C | A | 0.445 | -0.039 | 0.006 | 93067 | 46.096 |
|  | rs2228603 | T | C | 0.071 | -0.122 | 0.007 | 170510 | 311.084 |
|  | rs281393 | T | C | 0.373 | -0.032 | 0.006 | 93067 | 34.275 |
|  | rs8103315 | A | C | 0.136 | 0.042 | 0.006 | 156370 | 58.870 |
|  | rs75687619 | T | G | 0.024 | 0.159 | 0.015 | 91543 | 108.266 |
|  | rs6511720 | T | G | 0.098 | -0.185 | 0.006 | 184764 | 984.247 |
|  | rs386003 | T | G | 0.199 | 0.034 | 0.006 | 92419 | 35.176 |
|  | rs7412 | T | C | 0.066 | -0.374 | 0.010 | 92046 | 1514.474 |
|  | rs6016373 | G | A | 0.373 | -0.032 | 0.004 | 185730 | 78.518 |
|  | rs1800961 | T | C | 0.034 | -0.106 | 0.010 | 156406 | 110.561 |
|  | rs2277862 | T | C | 0.132 | -0.035 | 0.005 | 185738 | 45.044 |
|  | rs2235367 | G | A | 0.457 | 0.036 | 0.004 | 185691 | 104.039 |
|  | rs181360 | G | T | 0.199 | -0.028 | 0.004 | 178322 | 41.797 |
|  | rs138777 | G | A | 0.652 | -0.021 | 0.004 | 185274 | 33.452 |
|  | rs4253772 | T | C | 0.119 | 0.032 | 0.006 | 185188 | 30.821 |
|  |  |  |  |  |  |  |  |  |
| Smoke | rs653953 | A | G | 0.675 | -0.011 | 0.001 | 468170 | 56.563 |
|  | rs10458563 | G | A | 0.209 | 0.011 | 0.002 | 468170 | 43.974 |
|  | rs2391706 | T | C | 0.549 | -0.008 | 0.001 | 468170 | 31.478 |
|  | rs145589901 | T | C | 0.073 | 0.016 | 0.003 | 468170 | 34.913 |
|  | rs301805 | G | T | 0.582 | 0.008 | 0.001 | 468170 | 33.279 |
|  | rs1923216 | T | C | 0.625 | -0.010 | 0.001 | 468170 | 52.536 |
|  | rs12042107 | C | T | 0.549 | -0.009 | 0.001 | 468170 | 45.089 |
|  | rs34000440 | A | C | 0.217 | -0.009 | 0.002 | 468170 | 32.134 |
|  | rs4949465 | C | T | 0.130 | 0.011 | 0.002 | 468170 | 30.740 |
|  | rs10914684 | A | G | 0.325 | -0.009 | 0.001 | 468170 | 34.776 |
|  | rs11811176 | C | T | 0.073 | 0.015 | 0.003 | 468170 | 32.769 |
|  | rs1514174 | T | C | 0.565 | -0.009 | 0.001 | 468170 | 42.112 |
|  | rs11802893 | C | G | 0.121 | -0.014 | 0.002 | 468170 | 46.054 |
|  | rs10863810 | A | T | 0.217 | -0.010 | 0.002 | 468170 | 36.601 |
|  | rs2339515 | T | G | 0.381 | 0.009 | 0.001 | 468170 | 38.089 |
|  | rs10210512 | G | T | 0.420 | 0.008 | 0.001 | 468170 | 29.307 |
|  | rs72804575 | C | A | 0.050 | 0.020 | 0.003 | 468170 | 40.955 |
|  | rs6433901 | T | C | 0.750 | 0.009 | 0.002 | 468170 | 34.877 |
|  | rs1271272 | A | G | 0.312 | -0.008 | 0.001 | 468170 | 30.285 |
|  | rs7572027 | T | C | 0.813 | 0.011 | 0.002 | 468170 | 38.602 |
|  | rs62107261 | C | T | 0.048 | -0.018 | 0.003 | 468170 | 30.134 |
|  | rs10193706 | C | A | 0.523 | 0.014 | 0.001 | 468170 | 103.607 |
|  | rs528301 | A | G | 0.554 | 0.012 | 0.001 | 468170 | 71.056 |
|  | rs12465974 | C | T | 0.361 | -0.009 | 0.001 | 468170 | 36.500 |
|  | rs1427506 | G | C | 0.865 | -0.012 | 0.002 | 468170 | 35.335 |
|  | rs7567570 | C | T | 0.827 | 0.012 | 0.002 | 468170 | 47.127 |
|  | rs56059523 | C | T | 0.121 | 0.012 | 0.002 | 468170 | 32.872 |
|  | rs2710331 | C | T | 0.342 | 0.008 | 0.001 | 468170 | 33.179 |
|  | rs34495106 | G | A | 0.627 | -0.013 | 0.001 | 468170 | 78.851 |
|  | rs705219 | A | T | 0.887 | 0.016 | 0.002 | 468170 | 56.605 |
|  | rs4479577 | T | C | 0.481 | 0.008 | 0.001 | 468170 | 33.248 |
|  | rs12485709 | C | T | 0.387 | -0.010 | 0.001 | 468170 | 48.958 |
|  | rs9842947 | T | C | 0.673 | 0.009 | 0.001 | 468170 | 36.403 |
|  | rs62260755 | G | C | 0.222 | 0.010 | 0.002 | 468170 | 36.901 |
|  | rs12487411 | A | G | 0.471 | -0.008 | 0.001 | 468170 | 35.124 |
|  | rs4676964 | T | C | 0.511 | 0.008 | 0.001 | 468170 | 37.235 |
|  | rs9835772 | T | A | 0.244 | 0.009 | 0.002 | 468170 | 33.161 |
|  | rs58400863 | A | G | 0.341 | -0.009 | 0.001 | 468170 | 38.562 |
|  | rs7668995 | A | T | 0.290 | -0.012 | 0.002 | 468170 | 63.744 |
|  | rs72886316 | C | T | 0.136 | 0.012 | 0.002 | 468170 | 37.287 |
|  | rs72678864 | A | G | 0.172 | -0.013 | 0.002 | 468170 | 47.090 |
|  | rs17003752 | G | A | 0.140 | -0.011 | 0.002 | 468170 | 31.270 |
|  | rs4479668 | T | G | 0.419 | -0.008 | 0.001 | 468170 | 29.503 |
|  | rs10461104 | A | G | 0.634 | 0.008 | 0.001 | 468170 | 30.067 |
|  | rs72712556 | A | G | 0.327 | -0.009 | 0.001 | 468170 | 37.276 |
|  | rs986391 | A | G | 0.634 | -0.012 | 0.001 | 468170 | 74.671 |
|  | rs6893752 | G | A | 0.742 | -0.010 | 0.002 | 468170 | 36.979 |
|  | rs73123076 | C | T | 0.628 | 0.009 | 0.001 | 468170 | 37.044 |
|  | rs4957528 | C | A | 0.792 | 0.010 | 0.002 | 468170 | 33.653 |
|  | rs12517438 | G | T | 0.541 | 0.007 | 0.001 | 468170 | 29.290 |
|  | rs1559278 | C | T | 0.361 | -0.008 | 0.001 | 468170 | 32.813 |
|  | rs27003 | C | T | 0.695 | 0.009 | 0.001 | 468170 | 36.988 |
|  | rs329124 | G | A | 0.421 | -0.008 | 0.001 | 468170 | 32.510 |
|  | rs12213996 | A | G | 0.386 | 0.008 | 0.001 | 468170 | 30.983 |
|  | rs4839955 | A | C | 0.520 | -0.008 | 0.001 | 468170 | 35.345 |
|  | rs9487626 | T | C | 0.817 | -0.017 | 0.002 | 468170 | 86.702 |
|  | rs3818987 | T | C | 0.476 | -0.009 | 0.001 | 468170 | 38.349 |
|  | rs6963853 | A | G | 0.429 | 0.009 | 0.001 | 468170 | 39.555 |
|  | rs10233018 | G | A | 0.504 | 0.011 | 0.001 | 468170 | 60.543 |
|  | rs12333760 | C | T | 0.165 | -0.011 | 0.002 | 468170 | 35.238 |
|  | rs4730682 | C | T | 0.493 | -0.010 | 0.001 | 468170 | 50.192 |
|  | rs896777 | T | C | 0.562 | 0.008 | 0.001 | 468170 | 30.193 |
|  | rs2705608 | C | A | 0.643 | -0.010 | 0.001 | 468170 | 45.468 |
|  | rs6951574 | C | T | 0.468 | 0.009 | 0.001 | 468170 | 42.699 |
|  | rs1565735 | A | T | 0.203 | -0.019 | 0.002 | 468170 | 119.336 |
|  | rs13263909 | T | C | 0.681 | -0.008 | 0.001 | 468170 | 29.929 |
|  | rs1899896 | T | C | 0.297 | 0.009 | 0.002 | 468170 | 35.099 |
|  | rs12114218 | C | T | 0.505 | -0.008 | 0.001 | 468170 | 32.850 |
|  | rs11103667 | T | C | 0.192 | 0.010 | 0.002 | 468170 | 34.118 |
|  | rs4543592 | C | T | 0.480 | 0.009 | 0.001 | 468170 | 44.708 |
|  | rs1246292 | C | T | 0.679 | 0.009 | 0.001 | 468170 | 37.407 |
|  | rs4837631 | T | C | 0.450 | -0.008 | 0.001 | 468170 | 35.437 |
|  | rs1490320 | T | C | 0.105 | -0.013 | 0.002 | 468170 | 33.477 |
|  | rs13301073 | A | G | 0.368 | 0.010 | 0.001 | 468170 | 48.680 |
|  | rs3025316 | C | T | 0.115 | 0.016 | 0.002 | 468170 | 56.784 |
|  | rs11012750 | G | A | 0.308 | 0.008 | 0.001 | 468170 | 31.047 |
|  | rs1926030 | C | T | 0.390 | 0.014 | 0.001 | 468170 | 95.334 |
|  | rs12770479 | A | G | 0.404 | -0.008 | 0.001 | 468170 | 31.847 |
|  | rs2175207 | G | A | 0.160 | 0.012 | 0.002 | 468170 | 38.525 |
|  | rs9423279 | G | C | 0.657 | -0.010 | 0.001 | 468170 | 45.145 |
|  | rs3896224 | G | A | 0.414 | -0.009 | 0.001 | 468170 | 38.104 |
|  | rs11255908 | G | T | 0.256 | 0.011 | 0.002 | 468170 | 45.077 |
|  | rs2588978 | C | T | 0.519 | -0.011 | 0.001 | 468170 | 59.994 |
|  | rs35891966 | A | G | 0.071 | -0.015 | 0.003 | 468170 | 31.893 |
|  | rs2155646 | C | T | 0.388 | 0.019 | 0.001 | 468170 | 191.093 |
|  | rs2010921 | A | G | 0.316 | 0.010 | 0.001 | 468170 | 41.426 |
|  | rs56213534 | G | A | 0.301 | -0.009 | 0.001 | 468170 | 39.641 |
|  | rs2939756 | A | G | 0.478 | -0.010 | 0.001 | 468170 | 48.055 |
|  | rs6265 | T | C | 0.189 | -0.014 | 0.002 | 468170 | 63.088 |
|  | rs7977812 | G | T | 0.245 | 0.009 | 0.002 | 468170 | 30.520 |
|  | rs1109480 | A | G | 0.389 | -0.009 | 0.001 | 468170 | 38.819 |
|  | rs11066972 | T | C | 0.142 | -0.011 | 0.002 | 468170 | 33.326 |
|  | rs7969559 | G | A | 0.721 | -0.010 | 0.002 | 468170 | 41.743 |
|  | rs9576071 | C | T | 0.497 | -0.007 | 0.001 | 468170 | 29.682 |
|  | rs7333559 | A | G | 0.789 | -0.010 | 0.002 | 468170 | 34.199 |
|  | rs4899753 | G | T | 0.910 | 0.013 | 0.002 | 468170 | 30.692 |
|  | rs7155595 | C | A | 0.326 | 0.008 | 0.001 | 468170 | 30.353 |
|  | rs12886628 | C | G | 0.663 | 0.008 | 0.001 | 468170 | 33.333 |
|  | rs1381274 | T | C | 0.463 | 0.008 | 0.001 | 468170 | 34.376 |
|  | rs12433109 | A | G | 0.399 | 0.009 | 0.001 | 468170 | 36.597 |
|  | rs12910916 | T | C | 0.212 | 0.014 | 0.002 | 468170 | 69.577 |
|  | rs2017500 | A | G | 0.513 | 0.009 | 0.001 | 468170 | 42.171 |
|  | rs9646259 | G | A | 0.705 | 0.009 | 0.002 | 468170 | 31.987 |
|  | rs752894 | G | A | 0.324 | 0.008 | 0.001 | 468170 | 31.108 |
|  | rs11861214 | T | G | 0.216 | -0.011 | 0.002 | 468170 | 43.945 |
|  | rs4888444 | G | A | 0.047 | -0.019 | 0.003 | 468170 | 32.917 |
|  | rs1050847 | T | C | 0.575 | -0.008 | 0.001 | 468170 | 29.242 |
|  | rs3748387 | G | A | 0.351 | 0.010 | 0.001 | 468170 | 43.821 |
|  | rs4790874 | T | C | 0.530 | 0.009 | 0.001 | 468170 | 38.928 |
|  | rs11078713 | G | A | 0.418 | -0.008 | 0.001 | 468170 | 31.678 |
|  | rs1825733 | T | C | 0.156 | -0.011 | 0.002 | 468170 | 35.190 |
|  | rs4632203 | T | A | 0.264 | 0.009 | 0.002 | 468170 | 32.793 |
|  | rs12970816 | A | G | 0.395 | 0.009 | 0.001 | 468170 | 37.820 |
|  | rs71367545 | A | G | 0.210 | 0.011 | 0.002 | 468170 | 38.637 |
|  | rs17733784 | C | T | 0.377 | -0.009 | 0.001 | 468170 | 38.763 |
|  | rs76608582 | A | C | 0.047 | -0.024 | 0.003 | 468170 | 49.442 |
|  | rs6119897 | A | G | 0.239 | 0.010 | 0.002 | 468170 | 41.046 |
|  | rs3790286 | C | T | 0.543 | 0.008 | 0.001 | 468170 | 36.134 |
|  | rs77217252 | T | G | 0.127 | 0.012 | 0.002 | 468170 | 32.083 |
|  | rs762995 | G | A | 0.535 | -0.009 | 0.001 | 468170 | 38.995 |

**Supplementary Table 2. Antihypertensive medication use among adults by CHD status.**

| Group | N | Rate (%) | p value |
| --- | --- | --- | --- |
| CHD=Yes | 563 | 94.1 % | <0.001 |
| CHD=No | 4989 | 84.8 % | <0.001 |
